# An ALLO-1–associated network links mitochondrial quality control to functional recovery in structurally compromised muscle

**DOI:** 10.1038/s42003-026-11093-z

**Published:** 2026-10-06

**Authors:** Anwesha Sarkar, Lilla Biriczová, Natalia A. Szulc, Pankaj Thapa, Remigiusz Serwa, Wojciech Pokrzywa

**Affiliations:** 1 Laboratory of Protein Metabolism, International Institute of Molecular and Cell Biology in Warsaw, 4 Ks. Trojdena Str., Warsaw, Poland; 2 IMol Polish Academy of Sciences, M. Flisa 6, Warsaw, Poland

**Keywords:** Energy metabolism, Caenorhabditis elegans

## Abstract

Muscle function can recover before damaged contractile structures are fully repaired, but the mechanisms enabling this recovery remain poorly understood. Using a temperature-sensitive *Caenorhabditis elegans* model of UNC-45 dysfunction, in which impaired myosin chaperoning triggers muscle proteotoxic stress and reversible paralysis, we show that coordinated movement recovers while sarcomere disorganisation persists. Quantitative proteomics identified ALLO-1, a selective-autophagy receptor previously characterised for paternal organelle clearance during embryogenesis, as induced during recovery from UNC-45dependent muscle stress. Functional and proteomic analyses revealed an ALLO-1–associated regulatory network comprising IKKE-1, SIP-1, DIM-1 and CAR-1 that modulates mitochondrial stress responses and muscle recovery. Loss of ALLO-1 or its associated factors altered mitochondrial homoeostasis and delayed functional recovery, whereas muscle-specific ALLO-1a restoration improved recovery, preserved mitochondrial network integrity, and modulated mitochondrial turnover despite reduced respiratory capacity. These findings identify ALLO-1 as a regulator of mitochondrial stress adaptation that enables functional recovery before completing structural repair.

## Introduction

Recovery of muscle function following stress or injury is a multifaceted process involving both structural and non-structural determinants. Following eccentric contraction-induced muscle damage, force deficits and their recovery have been linked to alterations in excitation-contraction coupling and sarcoplasmic-reticulum Ca²⁺ handling^1–3^, redoxassociated changes in myofibrillar Ca²⁺ sensitivity^3^, and changes in motor-unit behaviour^4^. These observations indicate that contractile performance cannot be inferred from myofibrillar architecture alone. However, the cellular pathways that support functional recovery while structural abnormalities persist remain to be fully elucidated.

The nematode *Caenorhabditis elegans* provides a genetically tractable system to dissect such recovery mechanisms. Its body-wall muscles share core architectural features with vertebrate striated muscles, including repeating sarcomeres anchored at dense bodies and M-lines^5,6^. UNC-45 is a conserved myosin chaperone that cooperates with HSP70 and HSP90 to support myosin folding and thick-filament assembly^7^. *C. elegans* encodes a single UNC-45 protein, whereas vertebrates express two paralogues, UNC45A and the striated-muscle–enriched UNC45B^8^. The physiological relevance of this chaperone system is underscored by the finding that biallelic pathogenic variants in human UNC45B cause a progressive myopathy^9^. The temperature-sensitive *unc-45(m94)* allele in *C. elegans* causes paralysis and myofilament disorganisation at restrictive temperature, providing a tractable model of acute myosin-proteostasis failure^7,10–12^. Strikingly, as we show here, these animals regain coordinated movement after return to permissive conditions despite persistent sarcomeric abnormalities. This temporal dissociation between functional and structural recovery provides a model for identifying cellular pathways that restore motility in post-mitotic muscle.

Contractile stress also engages mitochondrial quality-control pathways. Acute inhibition of autophagy in skeletal muscle leads to the accumulation of depolarised mitochondria and increased oxidative stress during damaging eccentric contractions^4,13^, whereas Parkin is required for acute exercise-induced mitophagy and contributes to the maintenance of mitochondrial function and turnover in skeletal muscle^14,15^. However, whether mitochondrial quality control contributes to early functional recovery while sarcomeric abnormalities persist remains to be established.

To investigate this phenomenon, we performed quantitative proteomic profiling of *unc-45(m94)* animals during early recovery and identified ALLO-1 as a strongly upregulated protein. ALLO-1 was previously characterised in *C. elegans* embryos as a selective-autophagy receptor required for paternal mitochondrial elimination through allophagy. It binds the Atg8 homologue LGG-1 and is regulated by phosphorylation by IKK-E1, a member of the TBK1/IKKε kinase family^16^. More recent work demonstrated that ALLO-1 interacts with EPG-7/ATG-11 and promotes local recruitment of the UNC-51/ULK autophagy–initiation machinery to paternal mitochondria^17^. These studies established ALLO-1 as a receptor for embryonic allophagy, but its post-developmental functions in somatic tissues are largely unexplored.

Here, we identify a post-developmental role for ALLO-1 in adult body-wall muscle, where it supports mitochondrial network integrity and modulates stress-responsive mitochondrial processing. We further identify an ALLO-1–associated network comprising IKKE-1; SIP-1, a pH-regulated small heat-shock protein^18^; DIM-1, an immunoglobulin-superfamily protein required for body-wall muscle integrity^19^; and CAR-1/LSM14, a cytoplasmic mRNA-regulatory factor implicated in endoplasmic reticulum (ER) organisation and mitochondrial Ca²⁺ dynamics^20,21^. Together, our findings reveal an ALLO-1–associated network linking mitochondrial quality control to functional recovery before complete sarcomere repair.

## Results

### ALLO-1 facilitates functional recovery during incomplete structural repair

**Fig. 1:**
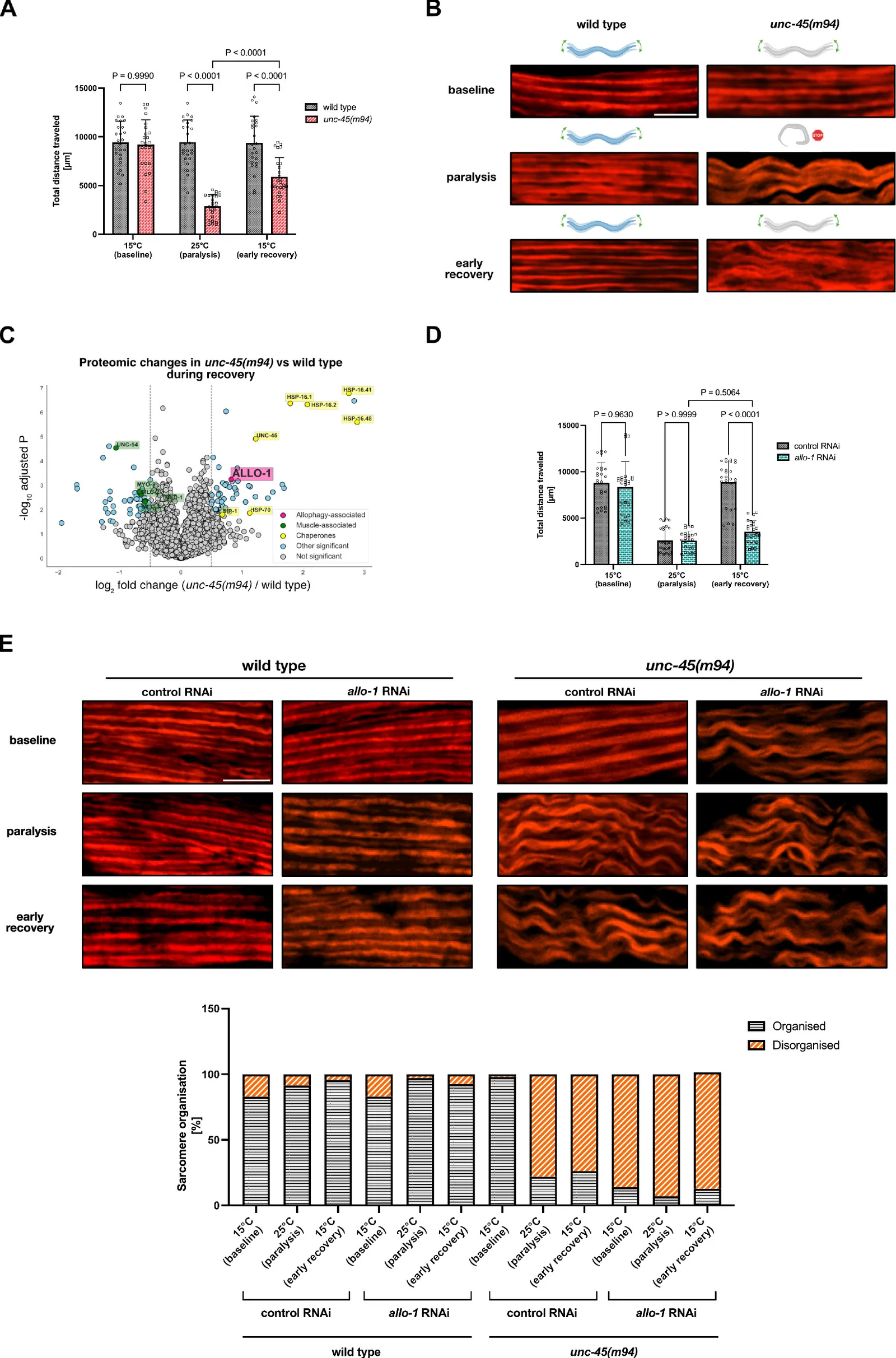
ALLO-1 facilitates functional recovery during incomplete sarcomere repair. **A** Quantification of motility in young adult wild-type (N2) and *unc-45(m94)* animals. Animals were maintained at 15 °C, shifted to 25 °C, and then allowed to recover at 15 °C for 1012 h. Motility was measured using the WormLab system as total distance travelled over time. Data represent mean ± SEM from from at least three independent biological replicates. Statistical analysis was performed using two-way ANOVA followed by Tukey’s multiple-comparisons test. **B** Representative confocal images of body-wall muscle stained with rhodamine-phalloidin to visualise F-actin organisation in young adult wild type and *unc-45(m94)* animals at 15 °C, 25 °C, and after early recovery at 15 °C. The scale bar shown in one representative image applies to all images. Scale bar, 5 µm. **C** Volcano plot from tandem mass tag-based quantitative proteomic analysis comparing wild-type and *unc-45(m94)* animals under recovery conditions. Three independent biological replicates were analysed per condition. Statistical significance was determined using a permutation-based two-sample *t*-test (S₀ = 0.1, FDR < 0.05). **D** Quantification of motility in young adult *unc-45(m94)* animals subjected to control (empty-vector) or *allo-1* RNAi. RNAi was initiated at the L4 stage. Animals were maintained at 15 °C, shifted to 25 °C, and then allowed to recover at 15 °C. Motility was measured using the WormLab system as total distance travelled over time. Data represent mean ± SEM from from at least three independent biological replicates. Statistical analysis was performed using two-way ANOVA followed by Šidák’s’s multiple-comparisons test. **E** Top - representative confocal images of body-wall muscle stained with rhodamine-phalloidin in young adult wild-type and *unc-45(m94)* animals subjected to control or *allo-1* RNAi. RNAi was initiated at the L4 stage. Animals were maintained at 15 °C, shifted to 25 °C, and then allowed to recover at 15 °C. The scale bar shown in one representative image applies to all images. Scale bar, 5 µm. Bottom - quantification of sarcomere organisation of body-wall muscle stained with rhodamine-phalloidin in young adult wild-type and *unc-45(m94)* animals subjected to control or *allo-1* RNAi. RNAi was initiated at the L4 stage. Animals were maintained at 15 °C, shifted to 25 °C, and then allowed to recover at 15 °C. Confocal microscopy images were classified into the categories organised (parallel, continuous sarcomeric actin bands with mostly preserved organisation, regular spacing and orientation) and disorganised (frequent discontinuities, substantial misalignment and loss of ordered structures) in a blinded scoring analysis. Data represent the percentage of images assigned to each category. For each condition, 3550 confocal images were analysed.

To investigate the mechanisms underlying functional recovery following proteotoxic muscle stress, we used the *C. elegans unc-45(m94)* model. At the permissive temperature of 15 °C, *unc-45(m94)* animals displayed coordinated locomotion and organised sarcomeres, whereas exposure to a restrictive temperature of 25 °C for 18–20 h resulted in paralysis and resulted in paralysis and visible myofilament abnormalities, including irregular, wavy filament organisation and loss of the normal parallel pattern (Fig. 1A, B). Following return to permissive conditions, the animals gradually recovered coordinated movement, with recovery becoming apparent after approximately 10–12 h (Fig. 1A). Notably, locomotor recovery occurred while myofilament abnormalities remained evident, indicating that muscle function can be restored before complete recovery of contractile architecture (Fig. 1B).

To identify proteins associated with this recovery phase, we performed quantitative proteomic profiling of wild-type (N2) and *unc-45(m94)* animals under three conditions: baseline at 15 °C, paralysis following exposure to 25 °C for 1820 h, and recovery for 10 h after return to 15 °C (Supplementary Data 1). During recovery, proteins increased in *unc-45(m94)* animals relative to wild type were enriched for processes related to protein folding, responses to temperature and heat, and actin-filament organisation, whereas proteins of reduced abundance were enriched for muscle contraction and muscle-system processes (Fig. S1A). These changes are consistent with persistent engagement of proteostasis pathways alongside incomplete restoration of the contractile apparatus. Exposure to the restrictive temperature increased the abundance of HSP-70 and several HSP-16 isoforms^22,23^, which remained elevated during recovery (Fig. 1C and Supplementary Data 1). In addition to this broad stress response, ALLO-1, a selective autophagy receptor previously characterised for its role in paternal mitochondrial elimination during *C. elegans* embryogenesis^16^, was induced during recovery (Fig. 1C and Supplementary Data 1). Although ALLO-1 was not among the most strongly induced proteins in the recovery proteomic dataset, it was prioritised because it represented a direct functional link between the proteostasis response triggered by UNC-45 dysfunction and selective regulation of organelles. Given that recovery occurred before complete restoration of sarcomere organisation, we reasoned that factors involved in regulated organelle processing could contribute to the early restoration of muscle function. We therefore initiated *allo-1* RNAi at the L4 stage to minimise developmental confounding and preferentially assess its post-developmental contribution to muscle recovery. Following exposure of *unc-45(m94)* animals to 25 °C and their subsequent return to 15 °C, *allo-1*-depleted worms displayed a pronounced delay in motility recovery compared with control worms (Fig. 1D). By contrast, RNAi depletion of several heat shock proteins induced during paralysis and recovery unexpectedly accelerated motility recovery (Fig. S1B). Thus, induction during proteotoxic stress does not necessarily imply a positive contribution to functional restoration, and ALLO-1 appears to have a distinct role in the recovery process.

To directly relate locomotor function to muscle architecture, animals from the same experimental cohort were divided between locomotor analysis and phalloidin-based assessment of myofilament organisation.wild-typetype animals, *allo-1* RNAi did not produce obvious alterations in the parallel organisation of sarcomeric filaments under the conditions examined (Fig. 1E). In *unc-45(m94)* mutants, however, *allo-1* depletion was associated with visible myofilament abnormalities already under permissive conditions, including irregular, “wavy” filament organisation and loss of the normal parallel pattern. Similar abnormalities remained evident during paralysis and early recovery (Fig. 1E). This pattern is consistent with a contribution of ALLO-1 to structural resilience in the sensitised *unc-45(m94)* background. Importantly, the structural abnormalities did not fully predict locomotor function. Under permissive conditions, *allo-1*-depleted *unc-45(m94)* animals remained motile despite detectable myofilament disorganisation, whereas following temperature-induced paralysis they showed delayed recovery relative to control animals (Fig. 1D, E). Thus, the presence of myofilament abnormalities was not by itself sufficient to abolish movement, suggesting that ALLO-1 supports functional recovery through mechanisms extending beyond the direct preservation of contractile architecture.

### ALLO-1 supports mitochondrial network integrity, particularly in the *unc-45(m94)* background

Because myofilament disorganisation did not fully account for the locomotor phenotype, we next asked whether ALLO-1 supports recovery through effects on mitochondrial homoeostasis. Given the established role of ALLO-1 in paternal mitochondrial elimination during embryogenesis, we examined mitochondrial network organisation in adult body-wall muscle. To visualise mitochondria, we used a muscle-specific TOMM-20::GFP reporter that labels the outer mitochondrial membrane^24^. Animals from the same experimental cohorts were divided between locomotor, sarcomere, and mitochondrial morphology analyses, enabling comparison of functional, contractile, and organellar phenotypes across matched experimental conditions (Fig. 1D, E). Mitochondrial organisation was quantified using a previously established image-analysis pipeline^25,26^, measuring mitochondrial area and the number of branch junctions per mitochondrion.

**Fig. 2:**
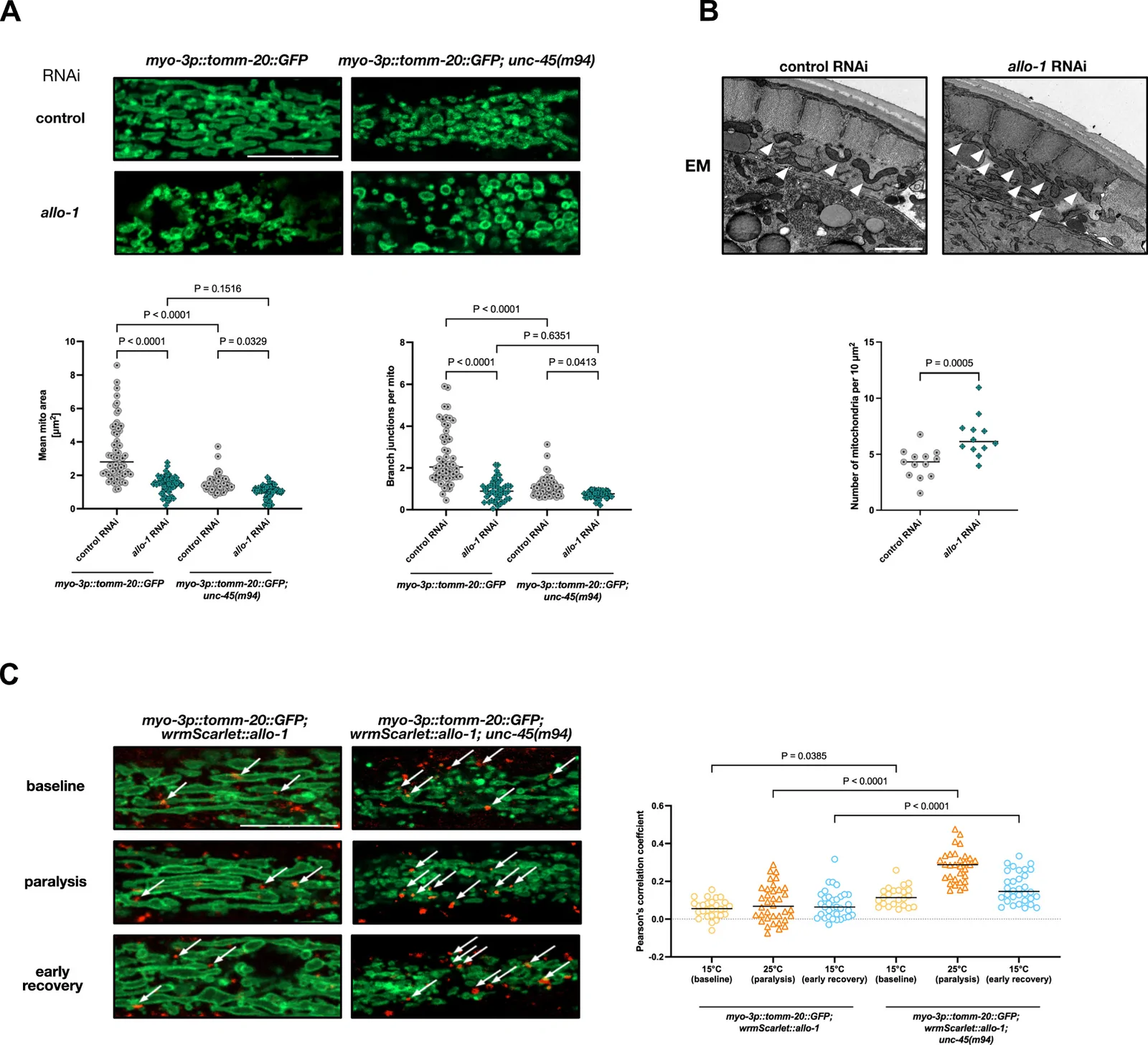
ALLO-1 supports mitochondrial network integrity in *unc-45(m94)* muscle. **A** Top - representative confocal images of young adult wild-type and *unc-45(m94)* animals expressing TOMM-20::GFP subjected to control or *allo-1* RNAi, initiated at the L4 stage, at 15 °C. The scale bar shown in one representative image applies to all images. Scale bar, 10 µm. Bottom - quantitative analysis of mitochondrial morphology parameters; mean mitochondrial area and number of branch junctions per mitochondrion. Mito - mitochondria. Data represent mean ± SEM from at least three independent biological replicates. Statistical significance was assessed by one-way ANOVA with Šidák’s multiple-comparisons test. **B** Top - representative transmission electron microscopy (EM) images of body-wall muscle mitochondria in young adult wild-type animals subjected to control or *allo-1* RNAi initiated at the L4 stage. White arrowheads indicate individual mitochondrial profiles. The scale bar shown in one representative image applies to all images. Scale bar, 2 µm. Bottom - quantification shows the number of mitochondrial profiles per 10 µm² of muscle-cell area. Measurements from 2 to 5 muscle cells were averaged for each animal and normalised to 10 µm² of muscle-cell area. Each point represents one animal; *n* = 14 animals per condition. Data represent mean ± SEM. Statistical analysis was performed using a two-tailed Mann-Whitney test. **C** Left - representative confocal images showing partial co-localisation of wrmScarlet::ALLO-1 (red) with TOMM-20::GFP-labelled mitochondria (green) in body-wall muscle of young adult wild-type and *unc-45(m94)* animals under normal conditions of 15 °C, during stress conditions of 25 °C and during early recovery conditions of 15 °C. White arrows indicate regions of overlap. The scale bar shown in one representative image applies to all images. Scale bar, 10 µm. Right - quantification of the degree of colocalization between GFP and wrmScarlet was performed using Pearson’s correlation coefficient. Dot plots represent data from 27 to 36 regions of interest (ROIs; individual cells). Statistical analysis was conducted using one-way ANOVA followed by Tukey’s multiple comparison test.

In wild-type animals, *allo-1* depletion significantly reduced both mean mitochondrial area and the number of branch junctions per mitochondrion, consistent with reduced mitochondrial network complexity and increased fragmentation (Fig. 2A). *unc-45(m94)* animals already displayed altered mitochondrial network organisation under permissive conditions, with reduced mitochondrial area and fewer branch junctions compared with wild type. Additional depletion of *allo-1* further reduced both parameters in the *unc-45(m94)* background (Fig. 2A), indicating that ALLO-1 contributes to maintenance of mitochondrial network organisation under conditions of compromised muscle proteostasis.

Transmission electron microscopy provided complementary ultrastructural evidence of altered mitochondrial organisation following *allo-1* depletion. Quantitative analysis revealed an increased density of mitochondrial profiles in muscle-cell sections (Fig. 2B). Together with the reduced mitochondrial area and branch-junction number detected by TOMM-20::GFP imaging, this finding is consistent with altered mitochondrial network organisation and increased fragmentation following ALLO-1 depletion.

To investigate the spatial relationship between ALLO-1 and mitochondria, we generated a CRISPR-engineered strain producing endogenously tagged wrmScarlet::ALLO-1 fusion protein. Treatment with *allo-1* RNAi markedly reduced wrmScarlet fluorescence, confirming the identity of the reporter signal and the effectiveness of the RNAi treatment (Fig. S2A). Confocal imaging detected wrmScarlet::ALLO-1 in body-wall muscle and revealed spatial overlap with TOMM-20::GFP-labelled mitochondria (Fig. 2C). Pearson’s correlation coefficients were generally higher in *unc-45(m94)* animals than in wild-type controls, with the strongest increase observed during temperature-induced paralysis at 25 °C. Following return to permissive conditions, the correlation decreased relative to the paralytic state but remained elevated, consistent with a stress-enhanced and partially persistent spatial association of ALLO-1 with mitochondria during recovery.

To determine whether the delayed recovery phenotype reflected broad physiological disruption rather than a selective effect on mitochondrial homoeostasis, we performed two additional control analyses. First, we examined polyQ40::YFP aggregation as a reporter of the tendency of muscle cells to accumulate aggregation-prone proteins^27^. Depletion of *allo-1* did not significantly alter the number of polyQ40::YFP aggregates in body-wall muscle (Fig. S2B), suggesting that loss of ALLO-1 does not cause a generalised increase in protein aggregation under the conditions examined. Second, because germline-derived GLP-1/Notch signalling can modulate somatic stress responses and organismal proteostasis^28–30^, we assessed whether *allo-1* depletion affected reproductive output. As expected, *glp-1* RNAi strongly reduced brood size, whereas *allo-1* RNAi had no significant effect (Fig. S2C), arguing against a major disruption of germline-dependent systemic stress regulation. Together, these control analyses indicate that the delayed recovery phenotype is unlikely to result from a broad collapse of proteostasis or general physiological dysfunction. Instead, they support a selective post-developmental role for ALLO-1 in mitochondrial homoeostasis during muscle recovery, consistent with the stress-enhanced association of endogenous ALLO-1 with mitochondria.

### ALLO-1–associated proteins differentially regulate mitochondrial organisation during recovery

**Fig. 3:**
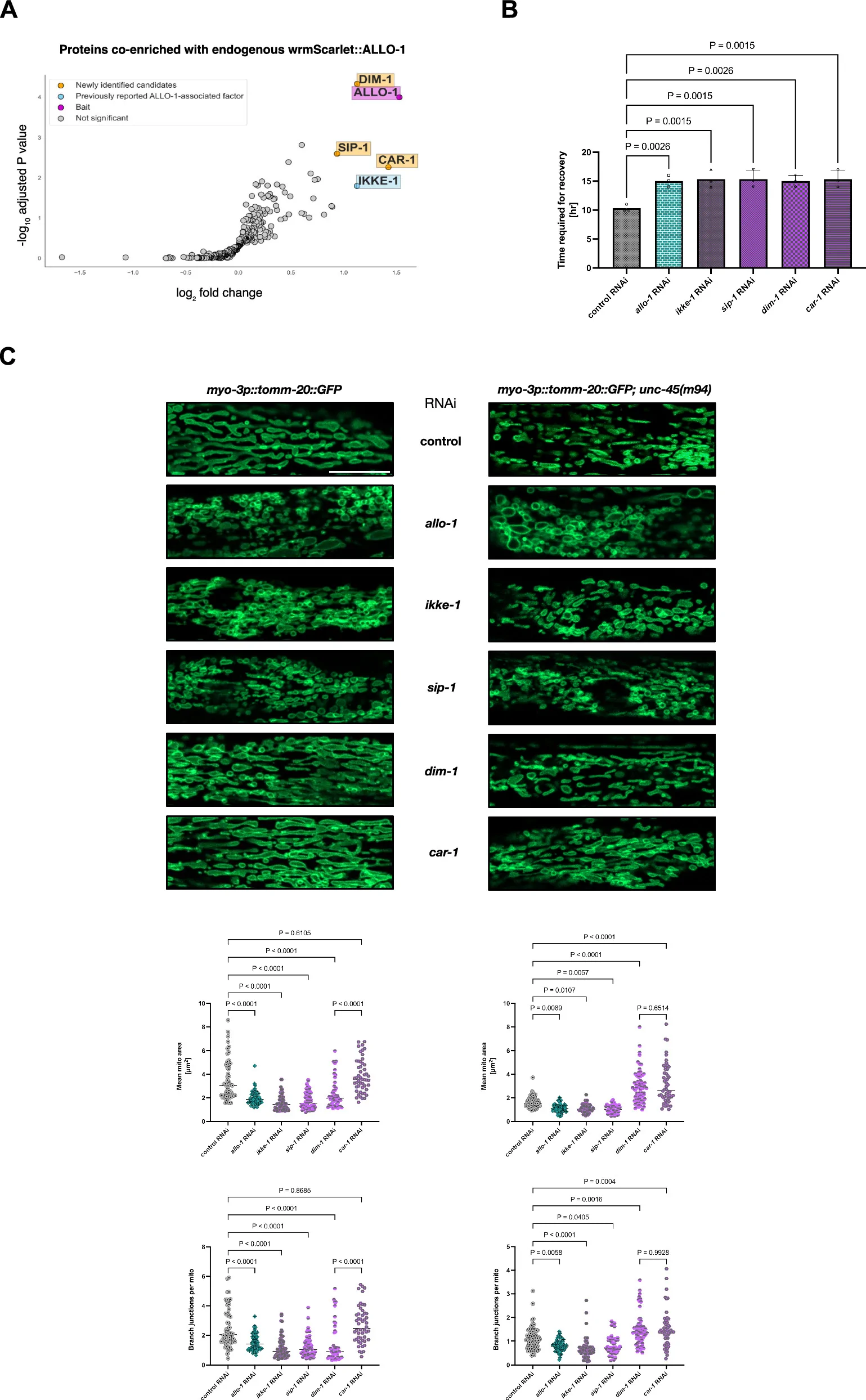
ALLO-1–associated proteins differentially regulate mitochondrial organisation during muscle recovery. **A** Volcano plot showing protein enrichment in wrmScarlet::ALLO-1 immunoprecipitates relative to the matched control. ALLO-1 is shown as the bait, IKKE-1 as a previously reported ALLO-1–associated factor, and SIP-1, DIM-1 and CAR-1 as newly identified candidate associated proteins. Lysates from young adult animals expressing endogenous wrmScarlet::ALLO-1 or untagged wild-type ALLO-1 (control) were processed in parallel using anti-RFP magnetic beads. Bound proteins were subjected to on-bead tryptic digestion, tandem mass tag labelling and liquid chromatography-tandem mass spectrometry analysis to identify proteins specifically enriched in wrmScarlet::ALLO-1 immunoprecipitates. Three independent biological replicates were analysed per genotype. Statistical significance was assessed using a permutation-based *t*-test (one-sided S₀ = 0.5; FDR = 0.01). Selected ALLO-1 associated proteins (IKKE-1, SIP-1, DIM-1 and CAR-1) are indicated. **B** Quantification of recovery time (hours required for the population to regain motility) for young adult *unc-45(m94)* animals after down-shift from 25 °C to 15 °C under control, *allo-1*, *ikke-1*, *sip-1*, *dim-1*, or *car-1* RNAi. RNAi was initiated at the L4 stage. Data represent mean ± SEM from ≥ 3 biological replicates. Statistical significance was determined by one-way ANOVA with Dunnett’s multiple-comparisons test. **C **Top - representative confocal images of body-wall muscle mitochondria in young adult wild-type and *unc-45(m94)* animals expressing *myo-3p::tomm-20::GFP* following control or gene-specific RNAi (*allo-1*, *ikke-1*, *sip-1*, *dim-1*, *car-1*), initiated at the L4 stage, at 15 °C. The scale bar shown in one representative image applies to all images. Scale bar, 10 µm. Bottom: quantitative analysis of mitochondrial morphology parameters. Mean mitochondrial area and number of branch junctions per mitochondrion. Mito - mitochondria. Data represent mean ± SEM from at least three independent biological replicates. Statistical significance was assessed by one-way ANOVA with Šidák’s multiple-comparisons test.

To identify proteins that may contribute to ALLO-1 function during muscle recovery, we immunoprecipitated endogenous wrmScarlet::ALLO-1 and analysed co-enriched proteins by comparative mass spectrometry. This approach identified both previously reported and previously unrecognised ALLO-1–associated proteins (Fig. 3A). Among the known factors, we detected IKK-1, a previously established regulator of ALLO-1 during embryonic allophagy^16^. We also identified SIP-1, a developmentally regulated small heat-shock protein whose chaperone activity is controlled by pH-dependent changes in oligomeric state^18^. SIP-1 had previously been detected as a candidate ALLO-1–associated protein^18^, and was also increased during muscle recovery in our proteomic dataset (Supplementary Data 2). To validate the interaction biochemically, endogenous wrmScarlet::ALLO-1 was immunoprecipitated and analysed by immunoblotting using a SIP-1–specific antibody. A faint SIP-1 signal was detected in wrmScarlet::ALLO-1 immunoprecipitates but not in parallel immunoprecipitates from untagged control animals (Fig. S3A), consistent with a weak and/or transient association between the two proteins. Because SIP-1 was previously described as being restricted to oocytes and embryos^18^, we examined its presence in adult muscle. Immunostaining detected SIP-1 immunoreactivity in body-wall muscle (Fig. S3B), supporting a post-developmental role for SIP-1 in the tissue where ALLO-1–associated mitochondrial regulation was analysed.

The ALLO-1–associated proteome additionally included DIM-1 and CAR-1, two proteins with distinct established cellular functions (Fig. 3A). DIM-1 is an immunoglobulin-superfamily protein containing three Ig-like domains that localises around dense bodies and is required for the structural integrity of body-wall muscle^19^. CAR-1/LSM14 is a cytoplasmic mRNA-regulatory factor that localises to P granules and other cytoplasmic ribonucleoprotein assemblies and has established roles in mRNA regulation, cytokinesis, and ER organisation^20,21^. CAR-1 has also been linked to mitochondrial Ca²⁺ dynamics during axon regeneration^20^. To examine whether these ALLO-1–associated proteins contribute functionally to muscle recovery, we initiated RNAi against *ikke-1, sip-1, dim-1*, or *car-1* at the early L4 stage in *unc-45(m94)* animals and monitored motility following temperature-induced paralysis. Depletion of each factor significantly delayed recovery compared with control RNAi, phenocopying the effect of *allo-1* depletion (Fig. 3B).

We next determined how depletion of these factors affected mitochondrial organisation. Consistent with the analyses presented in Fig. 2A, *unc-45(m94)* animals showed reduced mitochondrial area and network complexity already at the permissive temperature. Depletion of *allo-1, ikke-1*, or *sip-1* further reduced both parameters in wild-type and *unc-45(m94)* animals, supporting a role for these factors in maintaining mitochondrial network integrity (Fig. 3C). We also examined EPG-7/ATG-11, a scaffold of the FIP200/Atg11 family that links cargo recognition to autophagy initiation and is required for efficient ALLO-1–dependent allophagy during embryonic development^17^. Compared with depletion of *allo-1, ikke-1*, *sip-1, epg-7* RNAi produced relatively modest changes in mitochondrial area and branch-junction number, indicating that EPG-7 has a more limited effect on mitochondrial organisation in adult body-wall muscle under the conditions examined (Fig. S3C).

DIM-1 and CAR-1 showed a distinct, context-dependent pattern. Their depletion produced little or modest changes in mitochondrial morphology in wild-type animals but increased mitochondrial area and network complexity in the sensitised *unc-45(m94)* background, reversing the fragmentation phenotype (Fig. 3C). Notably, this apparent improvement in mitochondrial morphology was accompanied by delayed motility recovery (Fig. 3B, C), indicating that a more interconnected mitochondrial network does not necessarily predict improved muscle function. Together, these findings distinguish ALLO-1, IKKE-1, and SIP-1 as factors required to maintain mitochondrial network organisation, whereas DIM-1 and CAR-1 exert context-dependent effects on mitochondrial remodelling and recovery.

### ALLO-1–associated proteins modulate stress-responsive mitochondrial turnover in body-wall muscle

**Fig. 4:**
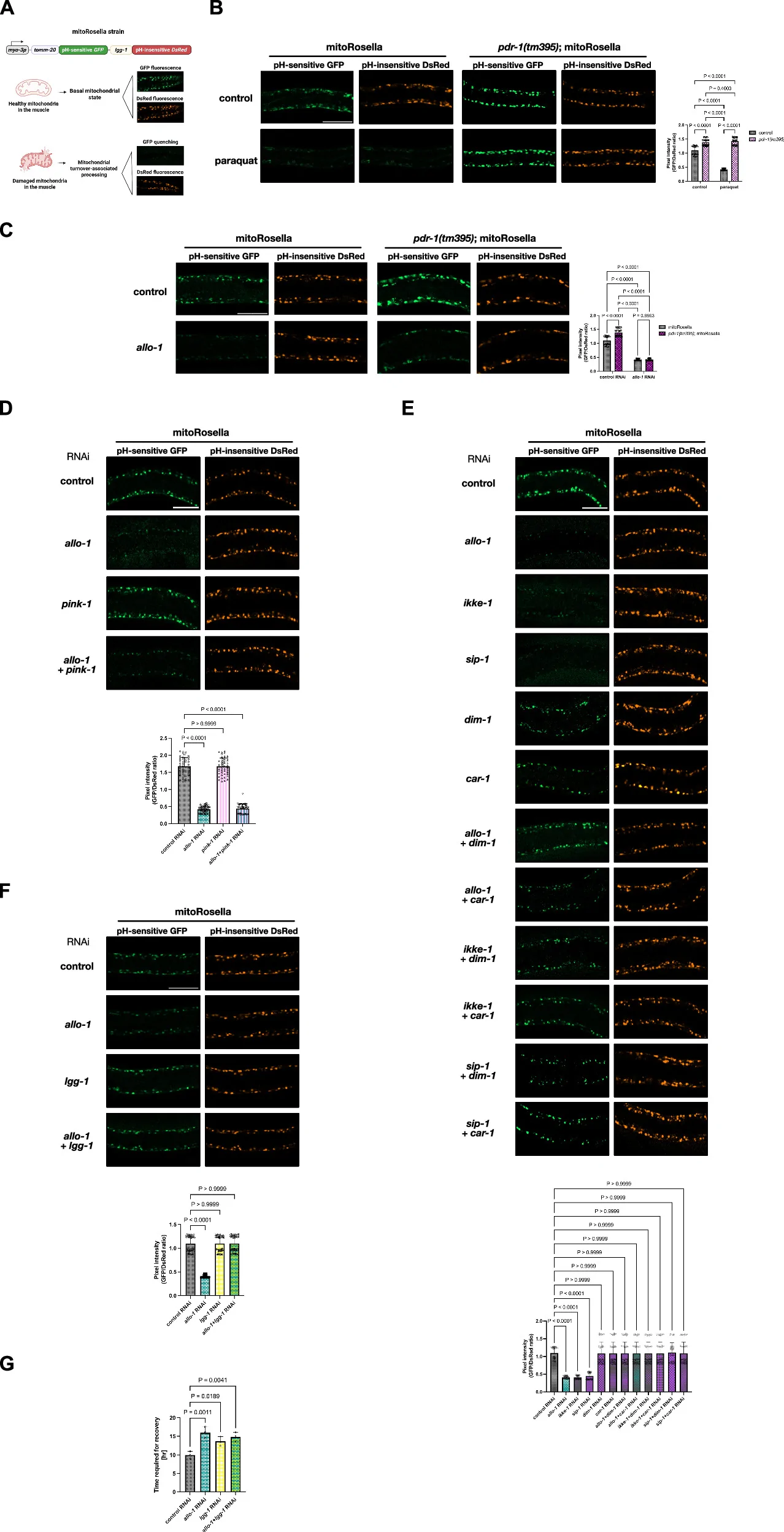
ALLO-1–associated proteins modulate stress-responsive mitochondrial turnover in body-wall muscle. **A** Schematic of the mitoRosella reporter used to monitor mitophagy. **B** Left - representative confocal images of body-wall muscle in young adult wild-type and *pdr-1(tm395)* animals expressing mitoRosella reporter subjected to paraquat initiated at the early L4 stage, at 20 °C. The scale bar shown in one representative image applies to all images. Scale bar, 50 µm. Right - quantification of the GFP/DsRed fluorescence-intensity ratio. Data represent mean ± SEM from from at least three independent biological replicates. Statistical significance was determined using two-way ANOVA with Tukey’s multiple-comparisons test. **C** Left - representative confocal images of body-wall muscle in young adult wild-type and *pdr-1(tm395)* animals expressing mitoRosella reporter subjected to control or RNAi targeting *allo-1* initiated at the early L4 stage, at 20 °C. The scale bar shown in one representative image applies to all images. Scale bar, 50 µm. Right - quantification of the GFP/DsRed ratio. Data represent mean ± SEM from from at least three independent biological replicates. Statistical significance was assessed by two-way ANOVA with Tukey’s multiple-comparisons test. **D** Top - representative confocal images of body-wall muscle in young adult animals expressing mitoRosella reporter subjected to control, *allo-1*, *pink-1* and *allo-1 + pink-1* RNAi initiated at the early L4 stage, at 20 °C. The scale bar shown in one representative image applies to all images. Scale bar, 50 µm. Bottom - quantification of the GFP/DsRed fluorescence-intensity ratio. Data represent mean ± SEM from from at least three independent biological replicates. Statistical significance was determined using one-way ANOVA with Šidák’s multiple-comparisons test. **E** Top - representative confocal images of body-wall muscle in young adult animals expressing mitoRosella reporter after control and single or combined *allo-1*, *ikke-1, sip-1, dim-1 and car-1* RNAi initiated at the early L4 stage, at 20 °C. The scale bar shown in one representative image applies to all images. Scale bar, 50 µm. Bottom - quantification of the GFP/DsRed fluorescence-intensity ratio, serving as a readout of mitophagic activity. Data represent mean ± SEM from from at least three independent biological replicates. Statistical significance was determined using one-way ANOVA with Dunett’s multiple-comparisons test. **F** Top - representative confocal images of body-wall muscle in young adult animals expressing mitoRosella reporter subjected to control, *allo-1*, *lgg-1* and *allo-1 + lgg-1* RNAi initiated at the early L4 stage, at 20 °C. The scale bar shown in one representative image applies to all images. Scale bar, 50 µm. Bottom - quantification of the GFP/DsRed fluorescence-intensity ratio. Data represent mean ± SEM from from at least three independent biological replicates. Statistical significance was determined using one-way ANOVA with Dunett’s multiple-comparisons test. **G** Quantification of recovery time (hours required for the population to regain motility) for young adult *unc-45(m94)* animals after down-shift from 25 °C to 15 °C under control, *allo-1*, *lgg-1*, *allo-1 + lgg-1* RNAi, initiated at the early L4 stage. Data represent mean ± SEM from from at least three independent biological replicates. Statistical significance was assessed using one-way ANOVA with Dunett’s multiple-comparisons test.

The identification of CAR-1, DIM-1, IKKE-1 and SIP-1 as ALLO-1–associated proteins suggested that these factors may converge on pathways regulating mitochondrial quality control. Because loss of ALLO-1 disrupted mitochondrial network integrity, we next examined whether ALLO-1 influences mitochondrial turnover in body-wall muscle. To this end, we used mitoRosella, a TOMM-20–targeted dual-fluorescence reporter comprising a pH-sensitive GFP and a pH-stable DsRed fluorophore. Under neutral conditions, both fluorophores are detected at mitochondria, whereas exposure of the reporter to acidic compartments selectively quenches GFP while preserving DsRed fluorescence (Fig. 4A). A reduction in the GFP/DsRed ratio therefore reports mitochondrial exposure to acidic environments and is commonly used as a proxy for mitophagy–associated mitochondrial processing^31^.

To validate the reporter under our experimental conditions, we exposed animals to paraquat, an inducer of oxidative mitochondrial stress known to activate PINK-1/PDR-1–dependent mitochondrial turnover^32^. Paraquat significantly reduced the mitoRosella GFP/DsRed ratio in wild-type animals, consistent with increased mitochondrial delivery to acidic compartments (Fig. 4B). By contrast, this response was absent in *pdr-1(tm395)* mutant animals, validating the stress responsiveness of the reporter and indicating that PDR-1 is required for paraquat-induced mitochondrial turnover under these conditions (Fig. 4B).

Having established that mitoRosella responds appropriately to mitochondrial stress in our system, we next examined whether ALLO-1 influences mitochondrial turnoverassociated processing. Depletion of *allo-1* by RNAi markedly reduced the mitoRosella GFP/DsRed ratio in body-wall muscle, consistent with increased mitochondrial exposure to acidic compartments (Fig. 4C). We next asked whether the mitochondrial turnoverassociated response induced by ALLO-1 depletion requires the canonical PINK-1/PDR-1 pathway. *allo-1* RNAi resulted in a decrease in the mitoRosella GFP/DsRed ratio in *pdr-1(tm395)* worms (Fig. 4C). Similarly, RNAi depletion of *pink-1* alone did not significantly alter the basal mitoRosella signal and did not prevent the response induced by *allo-1* depletion (Fig. 4D). Together, these findings indicate that ALLO-1–dependent mitochondrial turnover-associated processing does not require canonical PINK-1/PDR-1 signalling under the conditions tested and is consistent with engagement of a parallel mitochondrial quality-control pathway.

We next examined whether other ALLO-1–associated proteins influence this response. Depletion of *ikke-1* or *sip-1* phenocopied *allo-1* RNAi, producing a similar reduction in the mitoRosella GFP/DsRed ratio. By contrast, depletion of *dim-1* or *car-1* attenuated the response induced by *allo-1* RNAi (Fig. 4E). These genetic interactions mirror the distinct effects of these factors on mitochondrial morphology (Fig. 3C) and indicate that ALLO-1–associated proteins differentially modulate mitochondrial turnover-associated processing in a context-dependent manner.

Because mitoRosella reports mitochondrial exposure to acidic compartments, we next asked whether the response induced by ALLO-1 depletion requires the core autophagy machinery. Depletion of *lgg-1* prevented the reduction in the mitoRosella GFP/DsRed ratio caused by *allo-1* RNAi, indicating that this response depends on LGG-1–dependent autophagy machinery (Fig. 4F). To determine whether LGG-1 also contributes to functional recovery, we examined motility following temperature-induced paralysis. RNAi depletion of either *allo-1* or *lgg-1* significantly delayed recovery, whereas combined depletion did not produce a greater defect than either single treatment (Fig. 4G). These findings indicate that LGG-1–dependent autophagy contributes to both the mitochondrial processing response induced by ALLO-1 depletion and functional recovery following proteotoxic stress.

### Altered ALLO-1 levels influence mitochondrial state and muscle recovery

To validate the RNAi phenotypes and exclude sequence-specific off-target effects, we generated a CRISPR/Cas9 *allo-1* knockout (KO) strain. Quantitative analysis of TOMM-20::GFP-labelled mitochondria revealed reduced mitochondrial area and fewer branch junctions per mitochondrion in *allo-1* KO animals, closely recapitulating the fragmentation phenotype observed following *allo-1* RNAi (Fig. S4A). Additional depletion of *car-1, dim-1, ikke-1* and *sip-1* did not significantly modify these morphological parameters in the *allo-1* KO background (Fig. S4A). Thus, the contribution of individual ALLO-1–associated factors was no longer detectable following complete loss of ALLO-1. This result does not establish a requirement for residual ALLO-1 but instead indicates that the genetic effects of these factors depend on the perturbation context.

**Fig. 5:**
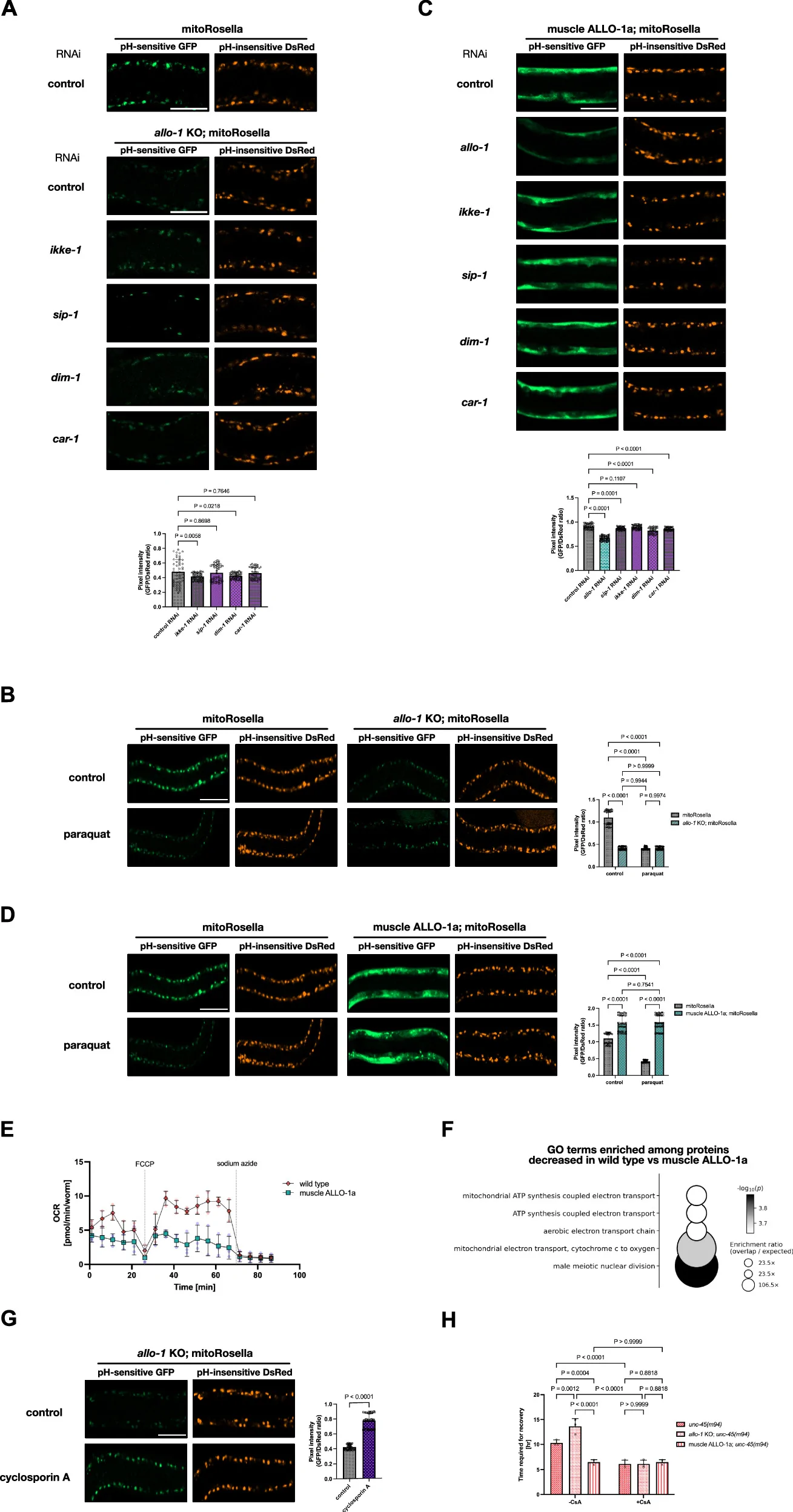
ALLO-1 loss and muscle-specific ALLO-1a expression differentially affect mitochondrial homoeostasis and functional recovery. **A** Top - representative confocal images of body-wall muscle mitochondria in *allo-1(syb10325)* knockout (KO) worms expressing the mitoRosella reporter, following control, *sip-1*, *dim-1*, *ikke-1* or *car-1* RNAi, initiated at the early L4 stage, at 20 °C. The scale bar shown in one representative image applies to all images. Scale bar, 50 µm. Bottom - quantification of the GFP/DsRed fluorescence-intensity ratios (*n* = 20 images per condition per biological repeat; three biological repeats). Data represent mean ± SEM and were analysed using one-way ANOVA with Dunnett’s multiple-comparisons test. **B** Left - representative confocal images of body-wall muscle in young adult wild-type and *allo-1(syb10325)* KO worms expressing mitoRosella reporter subjected to paraquat initiated at the early L4 stage, at 20 °C. The scale bar shown in one representative image applies to all images. Scale bar, 50 µm. Right - quantification of the GFP/DsRed fluorescence-intensity ratio, serving as a readout of mitophagic activity. Data represent mean ± SEM from from at least three independent biological replicates. Statistical significance was determined using two-way ANOVA with Tukey’s multiple-comparisons test. **C** Muscle ALLO-1a denotes animals carrying the integrated muscle-specific *myo-3p::FLAG-allo-1a::unc-54 3’UTR* transgene. Top - representative confocal images of body-wall muscle mitochondria in muscle ALLO-1a animals expressing the mitoRosella reporter, following the same RNAi treatments as in (A), at 20 °C. The scale bar shown in one representative image applies to all images. Scale bar, 50 µm. Bottom - quantification of the GFP/DsRed fluorescence-intensity ratios (*n* = 20 images per condition per biological replicate; three biological replicates). Data represent mean ± SEM. Statistical significance was determined by one-way ANOVA with Dunett’s multiple-comparisons test. **D** Left - representative confocal images of body-wall muscle in young adult wild-type and muscle ALLO-1a animals expressing mitoRosella reporter subjected to paraquat initiated at the early L4 stage, at 20 °C. The scale bar shown in one representative image applies to all images. Scale bar, 50 µm. Right - quantification of the GFP/DsRed fluorescence-intensity ratio, serving as a readout of mitophagic activity. Data represent mean ± SEM from from at least three independent biological replicates. Statistical significance was determined using two-way ANOVA with Tukey’s multiple-comparisons test. **E** Measurement of oxygen-consumption rates (OCR) in young adult wild-type and muscle ALLO-1a animals using a Seahorse XFp analyser, at 20 °C. Dark symbols and connecting lines represent the mean ± SD, while lighter symbols indicate individual replicate measurements (*n* = 3). FCCP carbonyl cyanide-4-(trifluoromethoxy)phenylhydrazone, an uncoupler of mitochondrial respiration, and sodium azide, a respiratory chain inhibitor used to block mitochondrial ATP production and assess non-mitochondrial oxygen consumption, were added at the indicated time points (vertical dashed lines). **F** Gene ontology (GO) biological process terms enriched among proteins downregulated in muscle ALLO-1a worms compared with wild type. Three independent biological replicates were analysed per genotype. Downregulated proteins were defined as those showing ≥ 0.3-fold decrease (S₀ = 0.1, FDR = 0.05). Enrichment analysis was performed in WebGestalt^52^ (FDR = 0.1, Benjamini-Hochberg method). Circle size denotes enrichment ratio; shading indicates statistical significance (-log₁₀ *P*). **G** Left - representative confocal images of body-wall muscle in young adult wild-type and *allo-1(syb10325)* KO worms expressing the mitoRosella reporter subjected to cyclosporin A (CsA) at the early L4 stage, at 20 °C. The scale bar shown in one representative image applies to all images. Scale bar, 50 µm. Right - quantification of the GFP/DsRed fluorescence-intensity ratio, serving as a readout of mitophagic activity. Data represent mean ± SEM from ≥ 3 biological replicates. Statistical significance was determined using a two-tailed Mann-Whitney test. **H** Quantification of recovery time (hours required for the population to regain motility) for young adult *unc-45(m94), allo-1* KO and muscle ALLO-1a animals expressing *unc-45(m94)* after down-shift from 25 °C to 15 °C, subjected to CsA, initiated at the early L4 stage. Data represent mean ± SEM from from at least three independent biological replicates. Statistical significance was assessed using two-way ANOVA with Tukey’s multiple comparisons test.

We next assessed the mitochondrial turnover-associated response using mitoRosella. Complete loss of *allo-1* caused a pronounced reduction in the GFP/DsRed ratio in body-wall muscle, consistent with the response observed following *allo-1* RNAi and indicating increased mitochondrial exposure to acidic compartments (Fig. 5A). To further examine the responsiveness of this altered reporter state to mitochondrial stress, we treated animals with paraquat. Paraquat reduced the GFP/DsRed ratio in control animals to a level comparable to that observed basally in *allo-1* KO animals, but did not produce a further detectable reduction in the KO background (Fig. 5B). Thus, complete loss of ALLO-1 results in a strong basal mitoRosella response that shows limited additional responsiveness to paraquat-induced mitochondrial stress. Additional depletion of *ikke-1* or *sip-1* did not further alter the reporter response in the *allo-1* KO background. Moreover, whereas depletion of *dim-1* or *car-1* attenuated the mitoRosella response caused by partial *allo-1* depletion, neither RNAi significantly modified the response following complete ALLO-1 loss (Fig. 5A). These observations indicate that the effects of ALLO-1–associated factors depend on the extent and context of ALLO-1 perturbation and do not support a simple linear hierarchy.

To complement these loss-of-function analyses, we generated an integrated transgenic strain expressing FLAG-tagged ALLO-1a specifically in body-wall muscle under the *myo-3* promoter (*myo-3p::FLAG-allo-1a::unc-54 3’UTR*). The *myo-3* promoter was also used to drive the TOMM-20::GFP and mitoRosella reporters, ensuring matched tissue specificity across assays. We selected ALLO-1a because it is an ALLO-1 isoform broadly conserved in nematodes and a well-characterized  functional model for ALLO-1–dependent selective autophagy. Although ALLO-1b shows preferential association with paternal mitochondria, ALLO-1a also localises to paternal mitochondria and has been characterised as an active autophagy receptor^17^. Expression of the integrated FLAG-ALLO-1a transgene was confirmed by anti-FLAG immunoblotting, which detected a band of the expected molecular mass that was markedly reduced following *allo-1* RNAi (Fig. S4B).

To further assess how increased ALLO-1 availability influences mitochondrial organisation, we first compared mitochondrial morphology in wild-type animals and animals expressing muscle-specific FLAG-ALLO-1a. Mean mitochondrial area was not significantly altered by ALLO-1a expression compared with wild-type animals (Fig. S4C), indicating that expression of the transgene itself does not induce a detectable basal mitochondrial morphology phenotype. We next examined whether depletion of individual ALLO-1–associated factors affects mitochondrial organisation in the ALLO-1a–expressing background. Quantitative analysis of TOMM-20::GFP-labelled mitochondria showed that depletion of *ikke-1*, *sip-1*, *dim-1*, or *car-1* did not significantly alter mitochondrial area or branch-junction numbers under these conditions (Fig. S4D). Thus, elevated ALLO-1a expression reduced the detectable contribution of individual ALLO-1–associated factors to mitochondrial morphology under the conditions tested.

We next examined whether increased ALLO-1a expression altered mitochondrial turnover-associated processing using mitoRosella. ALLO-1a–expressing animals displayed a diffuse GFP signal throughout body-wall muscle, whereas the DsRed signal remained punctate (Fig. 5C). This pattern was reduced following *allo-1* RNAi, confirming its dependence on the ALLO-1a transgene. Because the diffuse GFP distribution complicates interpretation of the GFP/DsRed ratio, we did not use the basal mitoRosella pattern alone as evidence for altered mitophagy. We therefore assessed mitoRosella responsiveness under mitochondrial stress. Paraquat induced the expected reduction in the mitoRosella GFP/DsRed ratio in control animals, whereas this response was markedly attenuated in ALLO-1a–expressing animals (Fig. 5D). These findings indicate that elevated ALLO-1a expression attenuates the mitoRosella response to mitochondrial stress, consistent with a role for ALLO-1 in regulating stress-responsive mitochondrial quality control.

We next asked whether the altered mitochondrial quality-control profile in ALLO-1a–expressing animals was accompanied by changes in respiratory output. To assess mitochondrial respiration, we measured whole-animal oxygen consumption at 20 °C using Seahorse extracellular flux analysis, an established approach for evaluating respiratory parameters in living *C. elegans*^33,34^. ALLO-1a–expressing animals exhibited reduced basal and maximal respiration compared with controls (Fig. 5E). Consistently, quantitative proteomic analysis revealed reduced abundance of proteins associated with oxidative phosphorylation and ATP synthesis (Fig. 5F and Supplementary Data 3). Despite this reduction in respiratory output, ALLO-1a–expressing animals maintained mitochondrial area comparable to control animals (Fig. S4C). These findings indicate that ALLO-1a expression uncouples mitochondrial network organisation from respiratory output. We next asked whether muscle-specific ALLO-1 function contributes to the functional recovery phenotype. To determine that we introduced the *myo-3p::FLAG-allo-1a* transgene into the *unc-45(m94); allo-1* KO background. Muscle-specific restoration of ALLO-1a significantly accelerated recovery compared with *unc-45(m94); allo-1* KO animals (Fig. S4E). These findings demonstrate that ALLO-1 activity within body-wall muscle is sufficient to promote recovery from UNC-45–associated muscle proteotoxic stress.

Finally, we asked whether pharmacological modulation of cyclophilin-sensitive mitochondrial processes could mitigate the functional consequences of ALLO-1 loss (KO). Cyclosporin A binds cyclophilins and is widely used to modulate processes associated with mitochondrial permeability transition. Because mitochondrial permeability transition has not been directly characterised in our system, we used cyclosporin A as a pharmacological probe of cyclophilin-sensitive mitochondrial responses rather than as a specific test of permeability-transition pore activity^35,36^. In *allo-1* KO animals, cyclosporin A significantly increased the mitoRosella GFP/DsRed ratio, partially reversing the altered basal reporter state associated with complete ALLO-1 loss (Fig. 5G). This finding indicates that cyclophilin-sensitive processes influence mitochondrial exposure to acidic compartments in the absence of ALLO-1. We next examined whether this modulation was accompanied by improved functional recovery. Cyclosporin A accelerated recovery of *unc-45(m94)* animals and improved the delayed recovery caused by ALLO-1 loss (Fig. 5H). By contrast, cyclosporin A provided no detectable additional benefit following muscle-specific restoration of ALLO-1a in the *allo-1* KO background (Fig. 5H). Consistently, cyclosporin A also improved the delayed recovery caused by *allo-1* RNAi (Fig. S4F). The lack of additivity between cyclosporin A treatment and muscle-specific ALLO-1a restoration suggests that these interventions may influence related mitochondrial stress-responsive processes, although these experiments do not establish a shared molecular mechanism. Together, these findings suggest that modulation of ALLO-1–dependent mitochondrial processing responses can influence functional recovery after proteotoxic muscle stress.

## Discussion

Our study identifies a previously unrecognised post-developmental function of ALLO-1 in adult body-wall muscle. Whereas ALLO-1 was originally characterised as a selective-autophagy receptor that promotes paternal organelle elimination during embryogenesis^16,17^, we found that it is strongly induced during early recovery from UNC-45–dependent proteotoxic stress and is required for timely restoration of motility. Both RNAi-mediated depletion and complete loss of ALLO-1 delayed recovery, whereas muscle-specific restoration of ALLO-1a substantially improved the phenotype, indicating that ALLO-1 activity within body-wall muscle is sufficient to promote recovery. Notably, *unc-45(m94)* animals began to regain coordinated movement while myofilament abnormalities remained evident, indicating that complete sarcomere repair is not a prerequisite for early functional recovery. The accompanying disruption of mitochondrial organisation following ALLO-1 loss implicates mitochondrial quality control as an important component of this recovery programme, although not necessarily its sole determinant.

The role of ALLO-1 in adult muscle is contextually distinct from its established function during embryonic allophagy. In embryos, IKKE-1–dependent phosphorylation of ALLO-1 promotes recruitment of LGG-1 and the autophagy-initiation machinery to paternal organelles^16,17^. In adult body-wall muscle, depletion of either *allo-1* or *ikke-1* similarly disrupted mitochondrial network organisation and enhanced mitochondrial exposure to acidic compartments. The response induced by *allo-1* depletion required LGG-1 but persisted following *pink-1* depletion and in *pdr-1* mutants, indicating that ALLO-1–dependent mitochondrial processing occurs independently of canonical PINK-1/PDR-1 signalling under the conditions tested. Conversely, elevated muscle ALLO-1a expression attenuated the paraquat-induced reporter response. Because mitoRosella detects mitochondrial exposure to acidic environments rather than mitophagic flux directly, these changes could reflect altered mitochondrial delivery, compartment acidification, lysosomal processing, or reporter handling. We therefore interpret ALLO-1 as modulating stress-responsive mitochondrial processing rather than as a direct inhibitor of mitophagy. Moreover, the discrete loss-of-function and transgenic-expression conditions used here do not establish a graded dose-response relationship.

SIP-1 adds a chaperone-associated component to this pathway. Previous work identified SIP-1 as a developmentally regulated small heat-shock protein whose chaperone activity is controlled by pH-dependent changes in oligomeric state^18^. We detected SIP-1 in endogenous ALLO-1 immunoprecipitates, observed its induction during recovery, and detected SIP-1 immunoreactivity in adult body-wall muscle. Depletion of *sip-1* phenocopied loss of *allo-1* and *ikke-1*, causing mitochondrial network fragmentation, enhancing the mitoRosella response associated with mitochondrial exposure to acidic compartments, and delaying functional recovery. These findings extend the function of SIP-1 beyond its previously characterised developmental context and implicate it in adult muscle mitochondrial homoeostasis. Local changes in pH, redox conditions, or protein-folding load during proteotoxic stress could modulate SIP-1 activity, although this possibility remains to be tested directly.

DIM-1 and CAR-1 displayed a different pattern of genetic interactions. DIM-1 is an immunoglobulin-superfamily protein required for body-wall muscle integrity^19^, whereas CAR-1/LSM14 is a cytoplasmic mRNA-regulatory factor with established roles in mRNA regulation, cytokinesis, ER organisation, and mitochondrial Ca²⁺ dynamics^20,21^. Depletion of either factor delayed recovery but partially restored mitochondrial area and network complexity in *unc-45(m94)* animals and attenuated the mitoRosella response caused by *allo-1* RNAi. Thus, a more interconnected mitochondrial network did not necessarily correspond to improved muscle function. This finding is consistent with evidence that mitochondrial remodelling can form part of an adaptive response to energetic or redox stress rather than serving solely as a marker of damage^31^. The effects of DIM-1 and CAR-1 were most apparent in the sensitised *unc-45(m94)* background and following *allo-1* RNAi, but were no longer detectable after complete ALLO-1 loss or in animals with elevated muscle ALLO-1a expression. Similarly, depletion of IKKE-1, SIP-1, DIM-1, or CAR-1 did not produce detectable changes in mitochondrial morphology in the ALLO-1a overexpression background. This lack of effect does not imply that these factors are dispensable, but rather suggests that their contribution becomes less detectable when ALLO-1 activity is increased. Thus, the contribution of individual components becomes apparent primarily when ALLO-1 function is limiting or mitochondrial homoeostasis is challenged. These context-dependent interactions argue against a simple linear hierarchy and instead support a non-linear regulatory network. DIM-1 may connect cytoskeletal organisation with mitochondrial remodelling, whereas CAR-1 may link post-transcriptional regulation with organelle physiology; however, the molecular basis of these relationships remains unresolved.

Elevated muscle ALLO-1a expression further revealed that preservation of mitochondrial architecture does not necessarily correspond to increased respiratory output. These animals maintained mitochondrial area and network complexity but exhibited reduced basal and maximal whole-animal oxygen consumption, accompanied by lower abundance of proteins associated with oxidative phosphorylation and ATP synthesis. The data therefore reveal a dissociation between mitochondrial morphology and respiratory capacity but do not establish that altered mitochondrial processing causes the respiratory phenotype. Because the oxygen-consumption measurements were performed at the whole-animal level, they also cannot determine whether the reduction originates exclusively from body-wall muscle. Direct measurements of ATP production, reactive oxygen species levels, mitochondrial membrane potential, and tissue-specific respiration will be required to define the functional consequences of ALLO-1 loss and elevated ALLO-1a expression. More broadly, the sensitivity of both sarcomeres and mitochondria to UNC-45 dysfunction may be relevant to human muscle disease, as biallelic variants in UNC45B cause progressive myopathy with eccentric cores^9^, although conservation of the ALLO-1–associated pathway in vertebrate muscle remains unknown.

The cyclosporin A experiments provide additional functional evidence connecting ALLO-1 deficiency with mitochondrial stress sensitivity. Cyclosporin A accelerated recovery in *unc-45(m94)* animals and improved the delayed recovery caused by *allo-1* RNAi or complete ALLO-1 loss. By contrast, it produced no detectable additional benefit following muscle-specific restoration of ALLO-1a. This lack of additivity suggests that cyclosporin A treatment and muscle-directed ALLO-1a expression may influence overlapping stress-protective mechanisms. However, cyclosporin A has multiple cyclophilin-dependent effects, and mitochondrial permeability transition has not been directly characterised in this system. The experiments therefore do not identify either the relevant cyclophilin target or a common molecular mechanism shared with ALLO-1.

Together, our findings identify ALLO-1 as a context-dependent regulator of mitochondrial quality control during muscle recovery. ALLO-1, IKKE-1, and SIP-1 support mitochondrial organisation and limit stress-responsive mitochondrial processing, whereas DIM-1 and CAR-1 exert distinct effects that become apparent in sensitised or partially compromised conditions. This non-linear network links mitochondrial homoeostasis with the restoration of muscle function before complete sarcomere repair and suggests that components of a developmental selective-autophagy pathway can contribute to adult tissue adaptation under proteotoxic stress.

## Methods

### *C. elegans* culture conditions

*C. elegans* strains were maintained on nematode growth medium (NGM) plates seeded with *Escherichia coli* OP50 or HT115, according to standard protocols^37^. Animals were cultured at 15 °C, 20 °C, or 25 °C, depending on the strain and experimental requirements.

### *C. elegans* strains used in the study

**Table:** 

Genotype	Source	Description
*C. elegans Bristol N2*	Caenorhabditis Genetics Centre (CGC)	Wild type
*unc-45(m94)*	Caenorhabditis Genetics Centre (CGC)	Temperature-sensitive *unc-45* mutant affecting myosin chaperone function
wacIs14[*myo-3p::tomm-20(1-50aa)::attB5::mGFP::unc-54 3’UTR, unc-119*(+)]	^24^	Integrated muscle-specific TOMM-20::GFP mitochondrial reporter
*myo-3p::mtRosella; unc-119*(+)	^31^	Muscle-specific mitoRosella mitochondrial processing reporter
*allo-1(syb8353*[wrmScarlet::*allo-1*])	SunyBiotech	Endogenously wrmScarlet-tagged ALLO-1
*allo-1(syb10325[allo-1(-1634bp*)])	SunyBiotech	N-terminal *allo-1* knockout allele
Is[*myo-3p::FLAG-allo-1a::unc-54 3’UTR, myo-*2p::gfp]	SunyBiotech (PHX8969)	Integrated muscle-specific FLAG-ALLO-1a transgene
wacIs14*[myo-3p::tomm-20(1-50aa)::attB5::mGFP::unc-54 3’UTR, unc-119*(+)]; wrmScarlet::ALLO-1	This study	wrmScarlet::ALLO-1 strain carrying integrated TOMM-20::GFP reporter
*unc-45(m94);* wacIs14[*myo-3p::tomm-20(1-50aa)::attB5::mGFP::unc-54 3’UTR, unc-119*(+)]; wrmScarlet::ALLO-1	This study	*unc-45(m94)* strain carrying wrmScarlet::ALLO-1 and TOMM-20::GFP reporter
Is[*myo-3p::FLAG-allo-1a::unc-54 3’UTR, myo-2p::gfp]; wacIs14[myo-3p::tomm-20(1-50aa)::attB5::mGFP::unc-54 3’UTR, unc-119*(+)]	This study	Muscle-specific FLAG-ALLO-1a transgene combined with integrated TOMM-20::GFP reporter
*myo-3p::mtRosella; Is[myo-3p::FLAG-allo-1a::unc-54 3’UTR, myo-2p::gfp]; unc-119(+)*	This study	Muscle-specific FLAG-ALLO-1a transgene combined with mitoRosella reporter
*allo-1(syb10325); unc-45(m94)*	This study	*allo-1* knockout combined with *unc-45(m94)* mutation
*allo-1(syb10325*); wacIs14[*myo-3p::tomm-20(1-50aa)::attB5::mGFP::unc-54 3’UTR, unc-119*(+)]	This study	*allo-1* knockout with integrated muscle TOMM-20::GFP reporter
*allo-1(syb10325); myo-3p::mtRosella*	This study	*allo-1* knockout with muscle mitoRosella reporter
*pdr-1(tm395)*	^38^	*pdr-1* mutant
*pdr-1(tm395);* myo-3p::mtRosella	This study	*pdr-1* mutant with muscle mitoRosella reporter
*unc-45(m94);* Is[*myo-3p::FLAG-allo-1a::unc-54 3’UTR, myo-2p::gfp]*	This study	*unc-45(m94)* strain carrying integrated muscle-specific FLAG-ALLO-1a transgene
Is[*myo-3p::FLAG-allo-1a::unc-54 3’UTR, myo-2p::gfp]; allo-1(syb10325); unc-45(m94)*	This study	*allo-1* knockout and *unc-45(m94)* strain carrying integrated muscle-specific FLAG-ALLO-1a transgene
rmIs133 [unc-54p::Q40::YFP]	CGC; AM141	Integrated body-wall muscle polyQ40::YFP aggregation reporter

### RNA interference

RNA interference (RNAi) was carried out using the standard feeding method, with bacterial clones sourced from the Ahringer *C. elegans* RNAi library^39^. NGM plates were supplemented with 1 mM IPTG (cat. IPT001; BioShop,) and 25 µg/ml carbenicillin (cat. 6344.2; Carl Roth GmbH & Co) and seeded with *E. coli* HT115 (DE3) carrying the L4440 plasmid containing the target gene insert. HT115 (DE3) bacteria transformed with the empty L4440 vector served as a negative control.

### Recovery assay for measurement of motility recovery time

Age-synchronised L4 hermaphrodites carrying the *unc-45(m94)* mutation were subjected to the RNAi treatments indicated in the corresponding figure legends. Animals were maintained at 15 °C before being shifted to 25 °C for 18–20 h to induce paralysis. Plates were then returned to 15 °C, and recovery of coordinated locomotion was monitored for approximately 10 h, or until the animals regained normal motility.

### Brood size assay

Age-synchronised L4 hermaphrodites of the wild-type strain were selected and subjected to RNAi treatments targeting *glp-1, allo-1* or a control RNAi (empty vector). For the assay, two worms were placed on each 35 mm NGM plate. Brood size measurements were conducted at a constant temperature of 20 °C. Worms were transferred to fresh NGM plates daily, from adulthood day 0 to adulthood day 4, marking the end of the egg-laying period. The number of eggs laid and larvae hatched were manually counted each day throughout the reproductive stage. For each biological repeat, eggs laid and larvae hatched from ten worms were counted. Data are presented as the total number of eggs laid and larvae hatched per plate, aggregated across three independent biological repeats.

### Cyclosporin A treatment

Age-synchronised L4 hermaphrodites were used for cyclosporin A (CsA) treatment assays. CsA (cat. 457970010; Thermo Fisher Scientific) was prepared in DMSO and applied at a final concentration of 50 µM by spot-seeding onto the bacterial lawn of NGM plates, as required for each experimental condition. Animals were maintained at the permissive temperature (15 °C) and subsequently transferred at L4 stage to CsA-containing plates, followed by incubation at the restrictive temperature (25 °C) for 1820 h to induce temperature-dependent paralysis. Following paralysis induction, plates were returned to 15 °C, and recovery of coordinated movement was monitored over time until animals regained motility.

### Paraquat treatment

Age-synchronised L4 hermaphrodites were used for paraquat-induced oxidative and mitochondrial stress assays. Paraquat (cat. 856177-1 G; Sigma-Aldrich) was prepared at a final concentration of 8 mM and added to heat-inactivated *E. coli* OP50 before seeding onto NGM plates. L4-stage animals were transferred onto paraquat-containing plates and maintained at 20 °C throughout the treatment period. Animals were imaged at the young adult stage using confocal microscopy to assess mitochondrial stress responses.

### Phalloidin staining

Sarcomere organisation was analysed by staining filamentous actin (F-actin) with Phalloidin-Atto 390 (cat. 50556; Sigma-Aldrich) following a modified protocol from^40^. Briefly, young adult *C. elegans* were washed off NGM plates seeded with *E. coli* OP50 using M9 buffer. The collected worms were snap-frozen in liquid nitrogen and dried in a SpeedVac concentrator at room temperature. Samples were permeabilised by incubation in 100% ice-cold acetone for 5 min. After complete acetone evaporation in a fume hood, worms were incubated with Phalloidin-Atto 390 for 30 min at room temperature in the dark to label F-actin. Stained animals were washed twice with M9 buffer containing 0.05% NP-40 (cat. NON505; BioShop) and 5 mg/mL BSA (cat. ALB001; BioShop) to remove unbound dye, then resuspended in a small volume of M9 and mounted on 2% (w/v) agarose pads. Coverslips were gently applied to avoid bubble formation, and samples were imaged using a Zeiss LSM 800 inverted confocal microscope equipped with a 63× oil-immersion objective.

### Immunostaining

Immunostaining was performed following protocols adapted from^41–44^ with minor modifications. Worms were cultured on NGM plates seeded with *E. coli* OP50 and collected at the young adult stage using M9 buffer. Samples were washed three times in M9 by centrifugation (1000 × *g*, 2 min) to remove bacteria, chilled on ice, and fixed in 500 µL of ice-cold formaldehyde solution for 15–30 min at room temperature. After fixation, worms were washed three times in 1× PBS-Tween (pH 7.2) and stored at 4 °C until further processing. For permeabilisation, samples were incubated overnight at 37 °C in β-mercaptoethanol (cat. 1610710; BioRad) solution with gentle mixing, followed by three washes in PBS-Tween. Collagenase (cat. C-5138 100 mg Collagenase IV; Sigma-Aldrich) treatment was then performed at 37 °C with vigorous shaking until ~20% of the animals appeared partially disrupted; the reaction was stopped by placing the tubes on ice. Worms were subsequently washed twice in PBS-Tween and once in antibody buffer (AbA). Primary antibody incubation was performed overnight at room temperature in AbA containing the antibody at a 1:250 dilution, with gentle agitation. The anti-SIP-1 antibody^18^ was used as the primary antibody. After incubation, worms were washed three times with AbA for 1 h each at room temperature or overnight at 4 °C. Secondary antibody (cat. A212452; Thermo Fischer Scientific) incubation was carried out in AbA at a 1:500 dilution for 1 h at room temperature in the dark, followed by three final washes in AbA. Worms were mounted on glass slides in antifade mounting medium, covered with a coverslip, and sealed with nail polish. Imaging was performed on a Zeiss LSM800 inverted confocal microscope equipped with a 63×/1.4 NA oil-immersion objective.

### Tandem mass tag-based quantitative proteomics

Unless otherwise stated, all tandem mass tag (TMT)-based quantitative proteomic analyses were performed using three independent biological replicates per condition. Protein extraction from *C. elegans* was performed using the Sample Preparation by Easy Extraction and Digestion (SPEED) protocol^45^. In brief, worm pellets were dissolved in concentrated trifluoroacetic acid (TFA; cat. T6508; Sigma-Aldrich) at a pellet-to-TFA ratio of 1:2-1:4 (v/v) and incubated for 2–10 min at room temperature. Samples were then neutralised with 2 M Tris-base buffer (10× the TFA volume) and incubated at 95 °C for 5 min in the presence of 10 mM Tris(2-carboxyethyl)phosphine and 40 mM 2-chloroacetamide. Protein concentrations were estimated by turbidity measurement at 360 nm, adjusted to equal levels using dilution buffer (2 M Tris-base/TFA, 10:1 v/v), and further diluted 1:4-1:5 with water. Proteins were digested overnight at 37 °C with trypsin (protein-to-enzyme ratio 100:1), and digestion was stopped by adding TFA to a final concentration of 2%. Peptides were labelled using an on-column TMT labelling approach^46^. Labelled samples were pooled into a single TMT multiplex, concentrated, and fractionated into 7-8 fractions using the Pierce High-pH Reversed-Phase Peptide Fractionation Kit (cat. 84868; Thermo Fisher Scientific). Fractions were reconstituted in 0.1% TFA, 2% acetonitrile prior to liquid chromatography-mass spectrometry analysis. Peptide separation was performed on an Easy-Spray Acclaim PepMap column (50 cm × 75 µm i.d.; cat. PN ES903; Thermo Fisher Scientific) at 55 °C using a 120-min gradient of acetonitrile in 0.1% formic acid at a flow rate of 300 nL min⁻¹. Mass spectrometric data were acquired on a Q Exactive HF-X Orbitrap instrument coupled to an UltiMate 3000 nano-LC via an Easy-Spray source (Thermo Fisher Scientific) operated in TMT mode. Full mass spectrometry (MS) scans were acquired at 60,000 resolution (m/z 200), followed by up to 15 data-dependent tandem mass spectrometry (MS/MS) scans of precursors (charge states 2-5; isolation window 0.7 m/z) fragmented by higher-energy collision dissociation (HCD, NCE 32). Dynamic exclusion was set to 35 s. Maximum injection times were 50 ms for MS and 96 ms for MS/MS (resolution 45,000, m/z 200). AGC targets were 3 × 10⁶ for MS and 1 × 10⁵ for MS/MS, with a minimum AGC of 1 × 10³. Raw data were processed in MaxQuant v1.6.17.0^47^ using the Andromeda search engine against the *C. elegans* reference proteome (UniProt ID: UP000001940). Reporter ion MS² quantification used a 0.003 Da mass tolerance and a minimum reporter PIF of 0.75. Modifications were set as follows: carbamidomethylation (C, fixed); oxidation (M, variable), deamidation (N/Q, variable), and N-terminal acetylation (variable). Trypsin/P was specified as the protease, allowing up to two missed cleavages. False discovery rates (FDRs) for peptides, proteins, and modification sites were set to 1%. “Match between runs” was enabled, and other parameters were kept at default settings. Quantification was based on unique and razor peptides, allowing protein grouping. Reporter intensity-corrected values were imported into Perseus v1.6.10.0^48^. Reverse hits, potential contaminants, and entries identified only by site were removed. Intensities were log₂-transformed, and only protein groups with complete quantitative data across all TMT channels were retained. Normalisation was performed by median subtraction within channels. Statistical significance was determined using a two-sample *t*-test with a permutation-based FDR of 0.05 and S₀  =  0.1 to identify differentially expressed proteins between conditions.

### Immunoprecipitation of ALLO-1

Immunoprecipitation of wrmScarlet::ALLO-1 was performed using RFP-Trap Magnetic Agarose (cat. rtma-20; ChromoTek GmbH), following the manufacturer’s protocol with minor adaptations for worm lysates. Young adult animals were collected from NGM plates using M9 buffer and washed three times by centrifugation (1000 × *g*, 2 min) to remove bacteria. Worm pellets were frozen in liquid nitrogen and stored at -80 °C until use. Frozen pellets were homogenised in ice-cold lysis buffer (10 mM Tris-HCl pH 7.5, 150 mM NaCl, 0.5 mM EDTA, 0.5% Nonidet P40 Substitute) supplemented with 1 mM PMSF and protease inhibitor cocktail. Homogenisation was performed using a chilled motorised pestle or bead beater until a uniform lysate was obtained. Lysates were incubated on ice for 30 min with gentle mixing and then clarified by centrifugation at 17,000 × *g* for 10 min at 4 °C. The supernatant was transferred to a pre-chilled tube and diluted 1:1 with ice-cold dilution buffer (10 mM Tris-HCl pH 7.5, 150 mM NaCl, 0.5 mM EDTA). For immunoprecipitation, 25 µL of RFP-Trap Magnetic Agarose slurry per sample was equilibrated by washing once with 500 µL dilution buffer. Diluted lysates were then added to the equilibrated beads and incubated for 1 h at 4 °C with gentle end-over-end rotation. Beads were then separated magnetically and washed three times with 500 µL wash buffer (10 mM Tris-HCl pH 7.5, 150 mM NaCl, 0.05% Nonidet P40 Substitute, 0.5 mM EDTA) with transferring the beads to a fresh tube during the final wash to minimise nonspecific carryover. For mass spectrometry-based proteomic analysis, bound proteins were additionally washed (3×) with a detergent-free buffer (10 mM Tris, 150 mM NaCl, 0.5 mM EDTA, pH 7.5) followed by an overnight incubation at 37 °C in 100 mM HEPES buffer pH 8.0 supplemented with 5 mM Tris(2-carboxyethyl)phosphine, 10 mM 2-chloroacetamide, and 1 µg sequencing grade trypsin. Following on-bead digestion, peptide solutions were separated from the beads and acidified with TFA to a final concentration of 1%. For Western Blot analysis. bound proteins were eluted by resuspending beads in 80 µL 2× SDS sample buffer (120 mM Tris-HCl pH 6.8, 20% glycerol, 4% SDS, 0.04% bromophenol blue, 10% β-mercaptoethanol) and heating for 5 min at 95 °C. Eluted proteins were analysed by SDSPAGE and Western blotting using an anti-mCherry antibody (cat. ab167453; Abcam).

### Tandem mass tag-based proteomic identification of ALLO-1 associated proteins

Following co-immunoprecipitation and on-bead digestion, the resulting tryptic peptides were labelled using an on-column TMT labelling protocol^46^. TMT-labelled samples were compiled into a single TMT sample and concentrated. Prior to LC-MS measurement, the peptide fractions were reconstituted in 0.1% TFA, 2% acetonitrile in water. Chromatographic separation was performed on an Easy-Spray Acclaim PepMap column 50 cm long × 75 µm inner diameter (Thermo Fisher Scientific) at 55 °C by applying 120 min acetonitrile gradients in 0.1% aqueous formic acid at a flow rate of 300 nl/min. An UltiMate 3000 nano-LC system was coupled to a Q Exactive HF-X mass spectrometer via an easy-spray source (all Thermo Fisher Scientific). The Q Exactive HF-X was operated in TMT mode with survey scans acquired at a resolution of 60,000 at m/z 200. Up to 18 of the most abundant isotope patterns with charges 2–5 from the survey scan were selected with an isolation window of 0.7 m/z and fragmented by higher-energy collision dissociation (HCD) with normalised collision energies of 32, while the dynamic exclusion was set to 35 s. The maximum ion injection times for the survey and MS/MS scans (acquired with a resolution of 30,000 at m/z 200) were 50 and 130 ms, respectively. The ion target value for MS was set to 3e6 and for MS/MS to 1e5, and the minimum AGC target was set to 1e3. The data were processed with MaxQuant v. 1.6.17.0^47^, and the peptides were identified from the MS/MS spectra searched against *C. elegans* reference proteome (UniProt ID: UP000001940) using the built-in Andromeda search engine. Reporter ion MS2-based quantification was applied with reporter mass tolerance = 0.003 Da and min. reporter PIF  =  0.75. Cysteine carbamidomethylation was set as a fixed modification, and methionine oxidation, glutamine/asparagine deamination, and protein N-terminal acetylation were set as variable modifications. For in silico digests of the reference proteome, cleavages of arginine or lysine followed by any amino acid were allowed (trypsin/P), and up to two missed cleavages were allowed. The FDR was set to 0.01 for peptides, proteins, and sites. Other parameters were used as pre-set in the software. Unique and razor peptides were used for quantification, enabling protein grouping (razor peptides are the peptides uniquely assigned to protein groups and not to individual proteins). Reporter intensity-corrected values were imported into Perseus v1.6.10.0^48^. Reverse hits, potential contaminants, and entries identified only by site were removed. Intensities were log₂-transformed, and only protein groups with complete quantitative data across all TMT channels were retained. Normalisation was performed by median subtraction within channels. PCA analysis performed on such processed data revealed that one of the control samples (ctrl_1) did not cluster well with the other two control samples (ctrl_2 and ctrl_3), plausibly due to an experimental error. The outlier control sample was excluded from analysis. ALLO-1 binding partners were defined as those meeting the threshold of a one-sided Student’s *t*-test (permutation-based FDR of 0.01 and S₀  =  0.5).

### SDS-PAGE and Western blotting

Protein samples were separated by SDS-PAGE on 10% polyacrylamide gels using a running buffer containing 25 mM Tris, 190 mM glycine, and 0.1% SDS. Electrophoresis was performed at a constant voltage of 120 V. Separated proteins were transferred onto PVDF membranes by wet transfer at 100 V for 1 h using a transfer buffer composed of 25 mM Tris, 190 mM glycine, and 10% methanol (pH 8.3). Total protein transfer was verified using the No-Stain Protein Labelling Reagent (cat. A44717; Thermo Fisher Scientific) according to the manufacturer’s instructions. Membranes were then blocked for 45 min at room temperature in 5% (w/v) skimmed milk prepared in TBST buffer (50 mM Tris-HCl, 150 mM NaCl, 0.1% Tween-20, pH 7.5). Blots were incubated overnight at 4 °C with the primary anti-mCherry antibody (cat. ab167453; Abcam) and anti-FLAG antibody (cat. F3165; Sigma-Aldrich) diluted in 5% milk/TBST. After three washes in TBST (10 min each), membranes were incubated for 1 h at room temperature with HRP-conjugated secondary antibodies at 1:10,000 dilution (cat. A0545; cat. A9044; Sigma-Aldrich) diluted in the same blocking buffer. Protein bands were visualised using enhanced chemiluminescence on a ChemiDoc Imaging System (Bio-Rad).

### Measurement of oxygen consumption rates (Seahorse XFp assay)

Oxygen consumption rates (OCR) were measured in *C. elegans* following a protocol adapted from^34^. Age-synchronised young adult wild-type and muscle ALLO-1a worms were collected from NGM plates seeded with *E. coli* OP50 and washed three times with M9 buffer by centrifugation (1000 × *g*, 2 min) to remove bacteria. Approximately 10–15 worms were transferred into each well of an eight-well Seahorse XFp cell culture microplate (Agilent) pre-coated with Cell-Tak (Corning) to ensure adherence during measurement. Basal OCR was recorded using a Seahorse XFp Analyser (Agilent) with a standard measurement cycle consisting of 3 min mixing, 2 min waiting, and 2 min measurement. Mitochondrial uncoupling was induced by injection of 10 µM FCCP (carbonyl cyanide-4-(trifluoromethoxy)phenylhydrazone; cat. SML2959; Sigma-Aldrich), and non-mitochondrial respiration was determined after subsequent injection of 40 mM sodium azide (cat. S2002; Sigma-Aldrich). OCR values were normalised to the number of worms per well.

### Worm motility assay

Age-synchronised young adult *C. elegans* wild type and *unc-45(m94)* mutant animals were cultured at 15 °C and then shifted to 25 °C for 18–20 h to induce paralysis. Subsequently, worms were returned to 15 °C for approximately 10 h to allow partial motility recovery. For motility analysis, animals from each condition were transferred to unseeded NGM plates and spontaneous movements were recorded for 2 min using the WormLab imaging system (MBF Bioscience). Imaging parameters, including frame rate (7.5 frames s⁻¹), exposure time, and gain, were kept constant across all recordings to ensure comparability. Movement trajectories were analysed in WormLab software (MBF Bioscience). Each assay included three independent biological replicates, with at least 75 animals analysed per replicate.

### PolyQ40::YFP aggregation assay

Age-synchronised young adult animals expressing polyQ40::YFP were maintained at 20 °C and imaged to quantify polyglutamine (polyQ) aggregation. Fluorescence images were acquired using an Axio Zoom.V16 stereomicroscope equipped with an Axiocam 705 monochrome CMOS camera (Carl Zeiss) under identical GFP exposure settings. The number of polyQ aggregates was quantified using ImageJ (NIH). Data represent measurements from 30 animals collected across three independent biological replicates.

### Confocal microscopy

High-resolution imaging of *C. elegans* samples was performed using a ZEISS LSM800 laser scanning confocal microscope (Carl Zeiss) equipped with 63×/1.4 NA oil-immersion and 10×/0.3 NA objectives. For all experiments, imaging parameters, including laser power, detector gain, and pixel size, were kept constant across samples to ensure quantitative comparability. Unless otherwise indicated, images correspond to single optical sections acquired under identical conditions. Image processing and analysis were carried out using ZEN (Zeiss) and ImageJ software. Only linear brightness and contrast adjustments were applied uniformly across the entire image for visualisation purposes without affecting the original data.

### Image analysis

Image analysis was performed using ImageJ (https://imagej.nih.gov/ij/). Fluorescence intensity was quantified as mean grey value within defined regions of interest (ROIs). Mitochondrial morphology was analysed using the ImageJ Fiji plugin “Mitochondria Analyser” v2.0.2 (https://sites.imagej.net/ACMito/). At least one ROI was analysed in a minimum of 20 images per condition across three biological replicates. For colocalization studies, the JaCoP plugin was applied^49^. Background subtraction was carried out using the rolling ball algorithm (radius = 50). Thresholds for the green and red channels were defined manually and maintained consistently across all images within a given experiment. Two ROIs containing individual cells per image were analysed in 13-18 images per condition. All brightness and contrast adjustments were applied uniformly and exclusively for visualisation, without altering quantitative results or interpretation.

### Transmission electron microscopy

Sample preparation and embedding for transmission electron microscopy (TEM) were performed as previously described^24^ with minor modifications. Briefly, *C. elegans* were immersed in 2.5% glutaraldehyde (EM grade; cat. G7651; Sigma-Aldrich) prepared in 0.1 M PBS (pH 7.5). While in the fixative, worms were carefully nicked with a fine needle to facilitate better penetration of the fixative and then incubated overnight at 4 °C. After fixation, samples were rinsed three times with PBS and post-fixed on ice in a mixture of 1% aqueous osmium tetroxide (Polysciences Europe GmbH and 1.5% potassium ferrocyanide (cat. P3289; Sigma-Aldrich) for 30 min. The specimens were then treated with 1% aqueous thiocarbohydrazide (cat. 88535; Sigma-Aldrich) for 40 min and post-fixed with 2% osmium tetroxide for 60 min at room temperature. Samples were subsequently incubated in 1% aqueous uranyl acetate (cat. 6159-44-0; CHMES) at 4 °C overnight, followed by staining with 0.66% lead aspartate for 60 min at 60 °C. After dehydration through a graded ethanol series, samples were infiltrated with epoxy resin (cat. 45-359; Sigma-Aldrich), placed between two Aclar sheets (cat. L4458; Agar Scientific) or positioned into flat silicone moulds to enable further animal cross-sections. Polymerisation was done at 60 °C for 72 h. Once the resin blocks were cured and the Aclar removed, samples were trimmed and sectioned using an ultramicrotome (EM UC7, Leica). Ultrathin sections (70 nm) were collected on 200-mesh nickel grids (cat. G2200N; Agar Scientific) or copper slot grids (cat. FCF 2010-CU-SB-50; Electron Microscopy Sciences). Sections were imaged using a Tecnai T12 BioTwin transmission electron microscope (FEI, Hillsboro, USA) equipped with a 16-megapixel TemCam-F416(R) camera (TVIPS GmbH).

### Statistics and reproducibility

All statistical analyses were performed using GraphPad Prism (version 11.1.0). Data are presented as mean ± SEM unless indicated otherwise. Statistical tests used for each experiment are specified in the corresponding figure legends and included one-way or two-way analysis of variance (ANOVA) followed by Tukey’s, Šidák’s or Dunnett’s multiple-comparisons tests, two-tailed Mann-Whitney tests, or Welch’s *t*-tests, as appropriate. Proteomic datasets were analysed using permutation-based *t*-tests with the indicated S₀ and FDR thresholds. GO enrichment analyses were performed using WebGestalt with Benjamini-Hochberg multiple-testing correction. Unless otherwise indicated, all experiments were independently repeated at least three times using biologically independent samples. Biological replicates were defined as independently prepared populations of *C. elegans* processed separately from culture through sample preparation and analysis. Technical replicates, when performed, are described in the relevant Methods sections. Sample sizes for each experiment are reported in the corresponding figure legends. For imaging experiments, each quantified data point generally represented an individual animal, cell, or rROI, depending on the assay. Pearson’s correlation coefficients for colocalization analyses were calculated from individual ROIs (cells). Mitochondrial morphology measurements were obtained from individual cells, whereas transmission electron microscopy analyses averaged measurements from multiple muscle cells per animal, with each animal treated as a single biological replicate. mitoRosetta analyses were performed on individual images collected across independent biological replicates. Motility assays were conducted on independent populations of animals, and proteomic analyses were performed using three independent biological replicates per condition. Representative microscopy images and immunoblots shown are representative of at least three independent biological experiments unless otherwise indicated. For qualitative assessment of sarcomere organisation, confocal images were scored in a blinded manner using predefined classification criteria. No statistical methods were used to predetermine sample size, and no data points or samples were excluded from the analyses unless predefined technical quality criteria were not met. Animals were allocated to experimental groups based on genotype and treatment, and experiments were performed under identical experimental conditions.

### Declaration of generative AI and AI-assisted technologies in the writing process

During the preparation of this work, the authors used ChatGPT to improve the readability and language of the manuscript. After using this tool, the authors reviewed and edited the content as needed and take full responsibility for the content of the published article.

## Supplementary information

Supplementary Information

Description of Additional Supplementary Files

Supplementary Data 1

Supplementary Data 2

Supplementary Data 3

reporting-summary

## Data Availability

The massspectrometry proteomics datasets generated during this study have been deposited in the ProteomeXchange Consortium^50^ via the PRIDE partner repository^51^ under the accession numbers PXD069274, PXD069277 and PXD069441. The raw and source data supporting the findings of this study, including data underlying the quantitative analyses and figures, are available in Zenodo at https://doi.org/10.5281/zenodo.17371038.
