# Supplementary material for: An ALLO-1–associated network links mitochondrial quality control to functional recovery in structurally compromised muscle
Source: Commun Biol. 2026 Oct 6;9:1348. doi: 10.1038/s42003-026-11093-z (PMC13669348; doi:10.1038/s42003-026-11093-z)

Fig S1

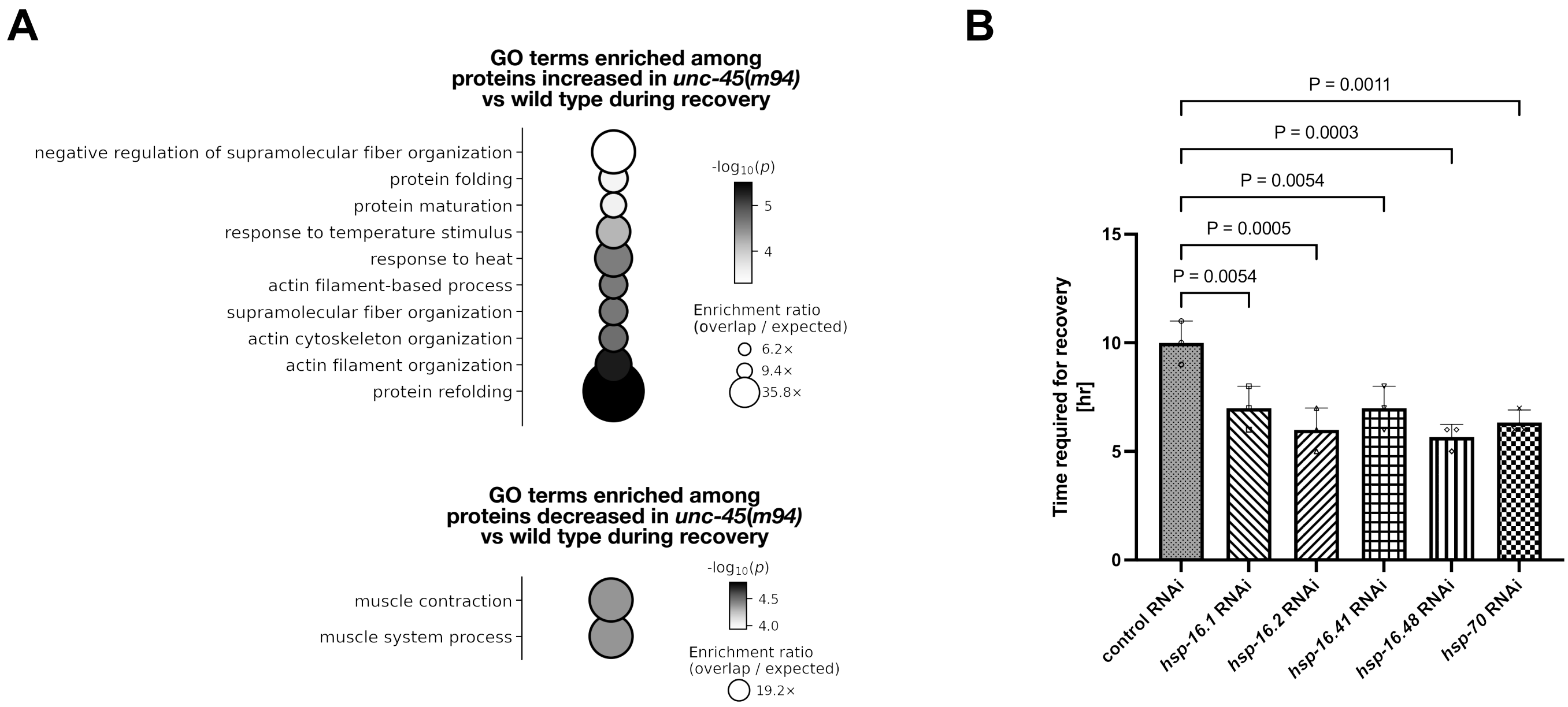

Figure S1. Proteomic and genetic analyses of functional recovery in unc-45(m94) animals. (A) Gene ontology (GO) enrichment analysis of biological process terms for proteins up- or down-regulated in *unc-45(m94)* animals during recovery. Up - and down-regulated proteins were defined as those showing ≥ 0.5-fold or ≤ -0.5 enrichment, respectively, relative to wild type in addition to meeting the initial permutation-based t-test criteria (two-sided, S_0 = 0.1, FDR = 0.05). GO analysis was performed using WebGestalt (FDR = 0.1, Benjamini-Hochberg method). Circle size denotes enrichment ratio; colour shading indicates statistical significance (-log10P). **(B)** Quantification of recovery time (hours required for of the population to regain motility) for young adult *unc-45(m94)* animals after down-shift from 25°C to 15°C under control (empty vector), *hsp-16.1*, *hsp-16.2*, *hsp-16.41*, *hsp-16.48*, *hsp-70*, or *allo-1* RNAi. RNAi was initiated at the early L4 stage. Data represent mean ± SEM from from at least three independent biological replicates. Statistical significance was assessed using one-way ANOVA with Dunnett's multiple-comparisons test.

Fig S2

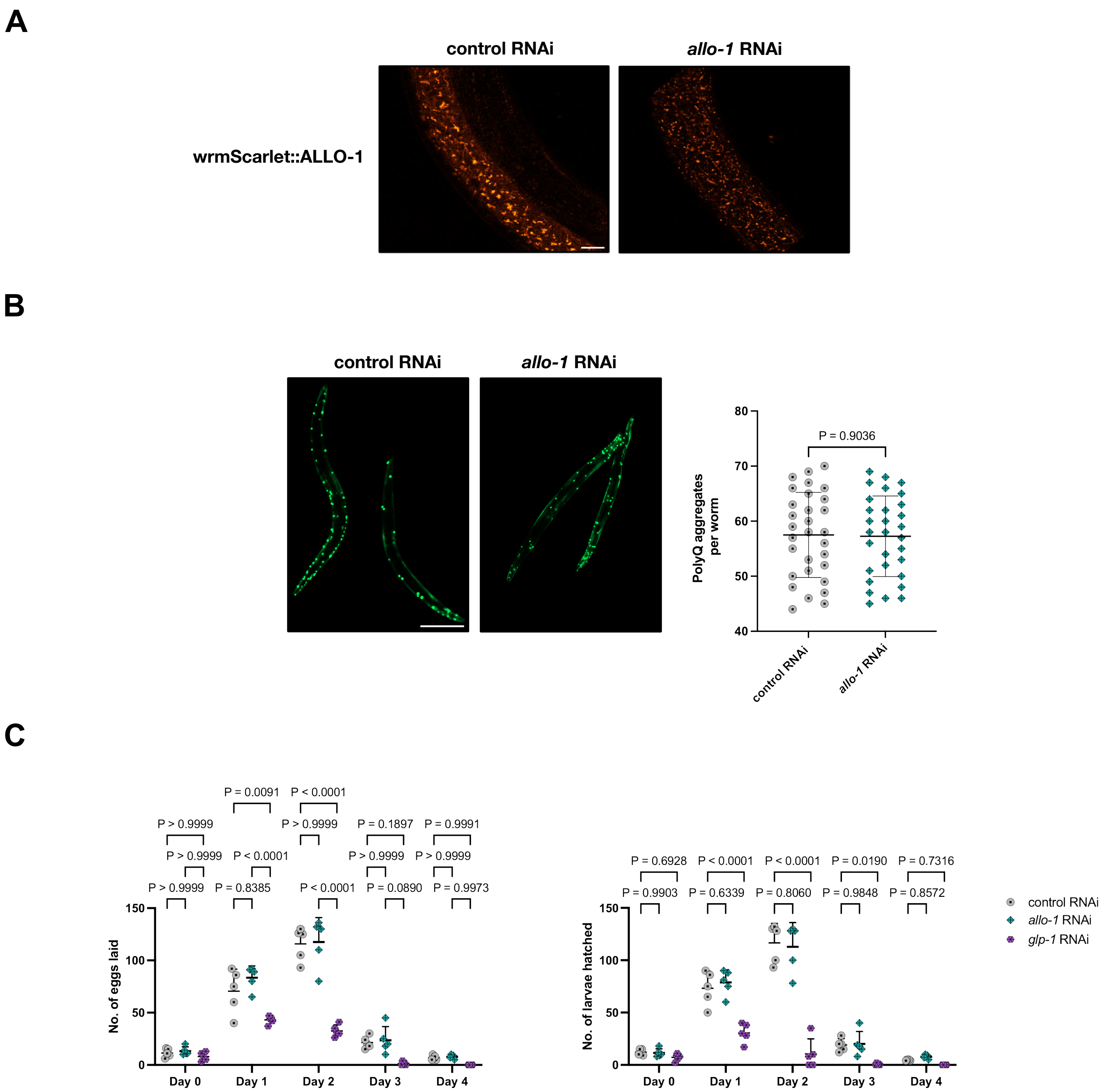

Figure S2. Validation of ALLO-1 reporter specificity and assessment of potential indirect effects. (A) Validation of *allo-1* RNAi specificity. Representative confocal images of wrmScarlet::ALLO-1 animals subjected to control or *allo-1* RNAi initiated at the early L4 stage, at 20°C. The scale bar shown in one representative image applies to all images. Scale bar, 10 µm. **(B)** Top - representative images of polyQ40::YFP aggregates in body-wall muscle following control or *allo-1* RNAi. The scale bar shown in one representative image applies to both images. Scale bar, 200 µm Bottom - quantification of polyQ40::YFP aggregates in body-wall muscle following control or *allo-1* RNAi. Each point represents one animal; horizontal lines indicate mean ± SD. Data represent $\geq$ 30 animals from three biological replicates. Statistical significance was assessed by the two-tailed Mann-Whitney test. **(C)** Assessment of fertility following *allo-1* or *glp-1* RNAi. Left - total number of eggs laid per day by wild type animals (two animals per plate) under control, *glp-1*, or *allo-1* RNAi initiated at the early L4 (n = 30 from three independent biological replicates). Right - total number of larvae hatched from the same plates, at 20°C. Data represent mean ± SEM. Statistical analysis used two-way ANOVA with Dunnett's or Tukey's multiple-comparisons test as indicated.

Fig S3

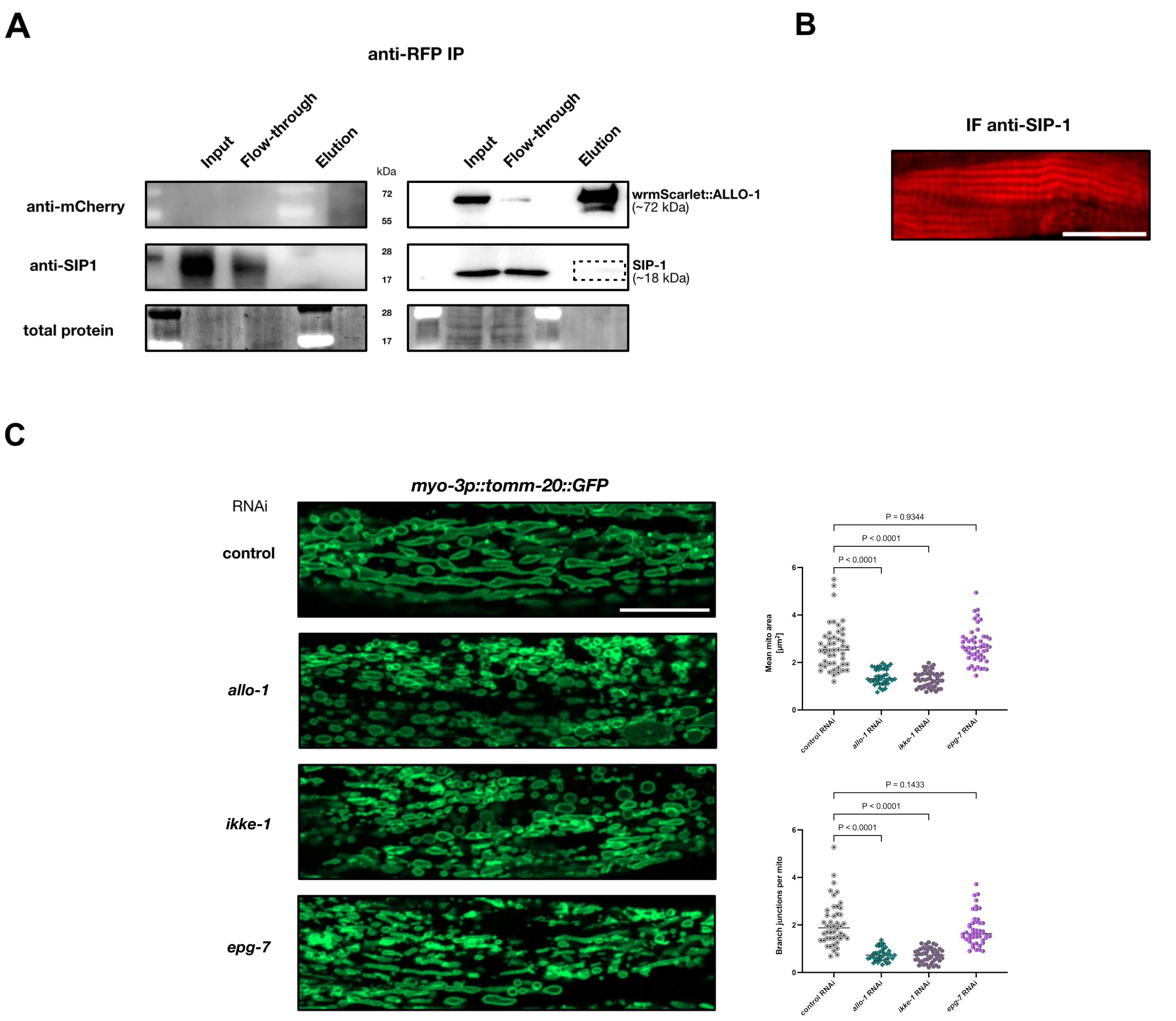

Figure S3. Orthogonal validation and functional analysis of ALLO-1-associated proteins. (A) Representative immunoblot showing enrichment of endogenous wrmScarlet::ALLO-1 following anti-RFP immunoprecipitation and detection of SIP-1 in the corresponding immunoprecipitate. A faint SIP-1 signal was detected in wrmScarlet::ALLO-1 pulldowns but not in immunoprecipitates prepared from untagged control animals. Immunoblots were probed with anti-mCherry and anti-SIP-1 antibodies. Total protein was visualized using No-Stain Protein Labeling Reagent. **(B)** Representative confocal image showing SIP-1 localization in body-wall muscle of young adult wild type animals stained with anti-SIP-1 antibody. The scale bar shown in one representative image applies to all images. Scale bar, 10 µm. **(C)** Top - representative confocal images of body-wall muscle mitochondria in young adult wild type animals expressing TOMM-20::GFP under control, *allo-1*, *ikke-1*, and *epg-7* RNAi, initiated at the early L4 stage, at 20°C. The scale bar shown in one representative image applies to all images. The scale bar shown in one representative image applies to all images. Scale bar, 10 µm. Bottom - quantitative analysis of mitochondrial morphology parameters in body-wall muscle of young adult wild type animals expressing TOMM-20::GFP after control, *allo-1*, *ikke-1* or *epg-7* RNAi initiated at early L4, at 20°C. Mean mitochondrial area and number of branch junctions per mitochondrion were quantified. Mito - mitochondria. Data represent mean ± SEM from at least three independent biological replicates. Statistical significance was assessed by one-way ANOVA with Dunett's multiple-comparisons test.

Fig S4

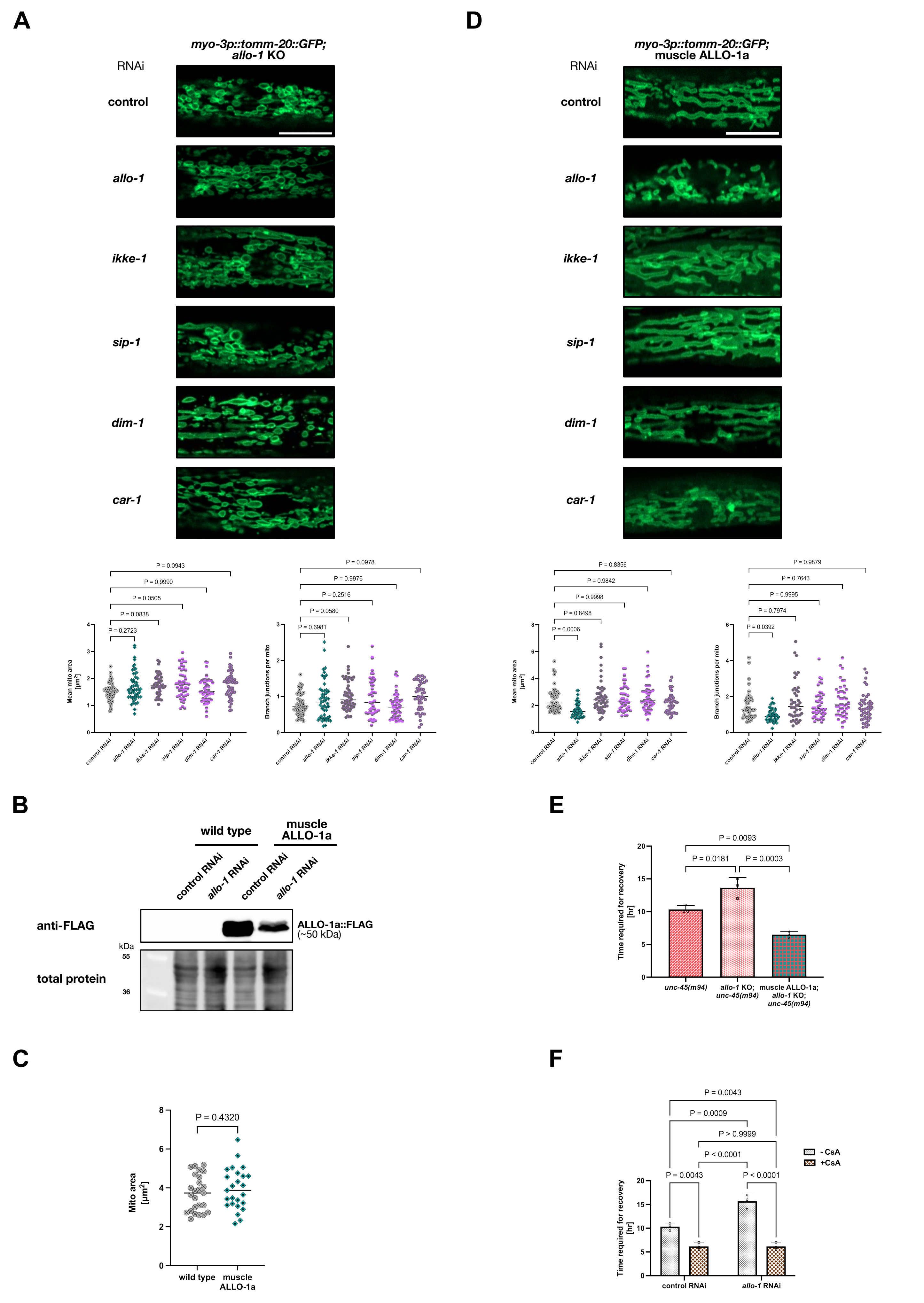

Figure S4. Mitochondrial morphology in ALLO-1 knockout and muscle-specific ALLO-1a expression backgrounds. (A) Top - representative confocal images of body-wall muscle mitochondria in young adult *allo-1* knockout (KO) animals expressing TOMM-20::GFP, following control, *allo-1*, *ikke-1*, *sip-1*, *dim-1*, or *car-1* RNAi, initiated at the early L4 stage, at 20°C. The scale bar shown in one representative image applies to all images. Scale bar, 10 µm. Bottom - quantitative analysis of mitochondrial morphology parameters in body-wall muscle of young adult *allo-1(syb10325)* KO worms expressing TOMM-20::GFP after control, *allo-1*, *ikke-1*, *sip-1*, *dim-1*, or *car-1* RNAi, initiated at the early L4 stage, at 20°C. Mean mitochondrial area and number of branch junctions per mitochondrion were quantified. Mito - mitochondria. Data represent mean ± SEM (n ≥ 3 biological replicates). Statistical significance was assessed by one-way ANOVA with Tukey's multiple-comparisons test. **(B)** Representative immunoblot showing reduced FLAG signal in muscle ALLO-1a animals following *allo-1* RNAi. Immunoblots were probed with an anti-FLAG antibody. Total protein loading was visualized using No-Stain Protein Labeling Reagent. **(C)** Quantitative analysis of mitochondrial area in body-wall muscle of young adult wild type and muscle ALLO-1a animals expressing TOMM-20::GFP initiated at L4, at 20°C. Mito - mitochondria. Data represent mean ± SEM from at least three independent biological replicates. Statistical significance was assessed by two-tailed Welch's t-test. **(D)** Top - representative confocal images of body-wall muscle mitochondria in young adult muscle ALLO-1a animals expressing TOMM-20::GFP, following control, *allo-1*, *ikke-1*, *sip-1*, *dim-1*, and *car-1* RNAi, initiated at the early L4 stage, at 20°C. The scale bar shown in one representative image applies to all images. Scale bar, 10 µm. Bottom - quantitative analysis of mitochondrial morphology parameters in body-wall muscle of young adult muscle ALLO-1a animals expressing TOMM-20::GFP after control, *allo-1*, *ikke-1*, *sip-1*, *dim-1*, or *car-1* RNAi, initiated at the early L4 stage, at 20°C. Mean mitochondrial area and number of branch junctions per mitochondrion were quantified. Mito - mitochondria. Data represent mean ± SEM from at least three independent biological replicates. Statistical significance was assessed by one-way ANOVA with Tukey's multiple-comparisons test. **(E)** Quantification of recovery time (hours required for the population to regain coordinated motility) in young adult *unc-45(m94)* animals carrying the indicated *allo-1* genotypes or the muscle-specific ALLO-1a transgene following downshift from 25°C to 15°C. Cyclosporin A (CsA) treatment was initiated at the early L4 stage. Data represent mean ± SEM from ≥ 3 biological replicates. Statistical analysis used one-way ANOVA with Šidák's multiple-comparisons test. **(F)** Quantification of recovery time (hours required for the population to regain coordinated motility) in young adult *unc-45(m94)* animals subjected to control or *allo-1* RNAi following downshift from 25°C to 15°C. CsA treatment was initiated at the early L4 stage. Data represent the mean ± SEM from at least three independent biological replicates. Statistical significance was determined by two-way ANOVA followed by Tukey's multiple comparisons test.

Uncropped immunoblots and total-protein loading controls corresponding to Supplementary Fig. S3A

anti-mCherry

wild type

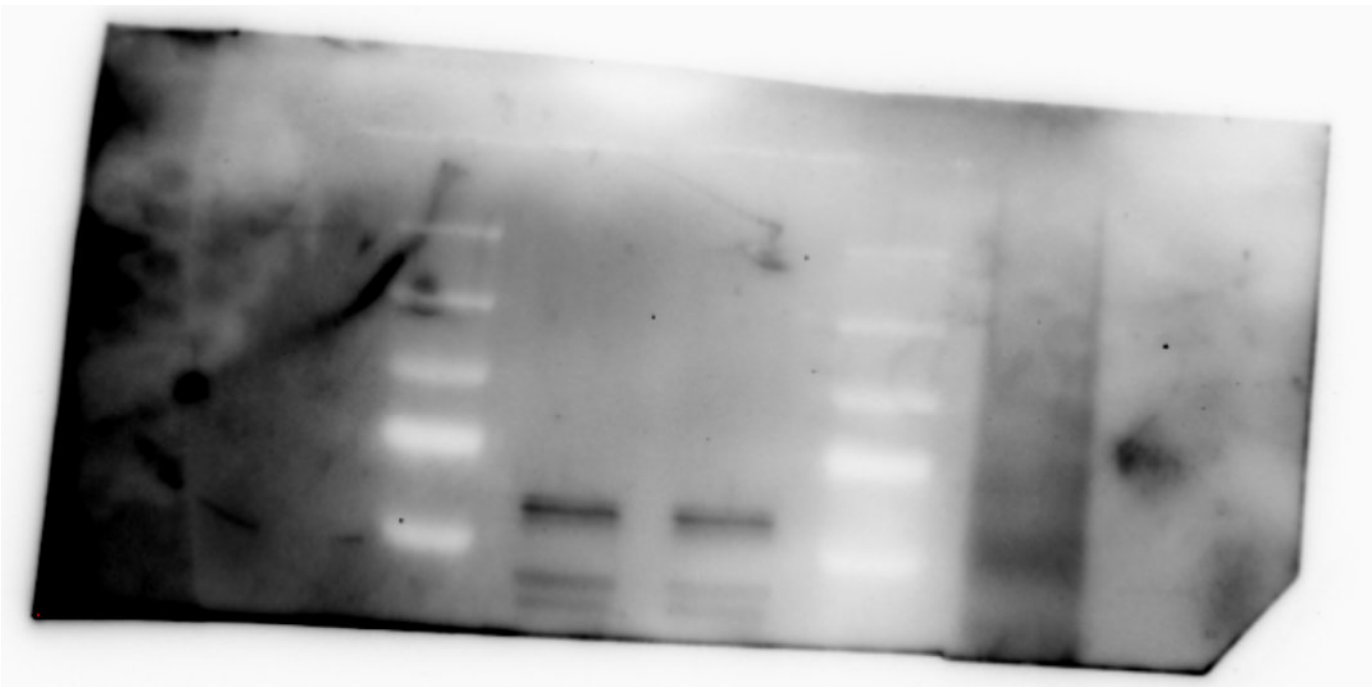

wrmScarlet::ALLO-1

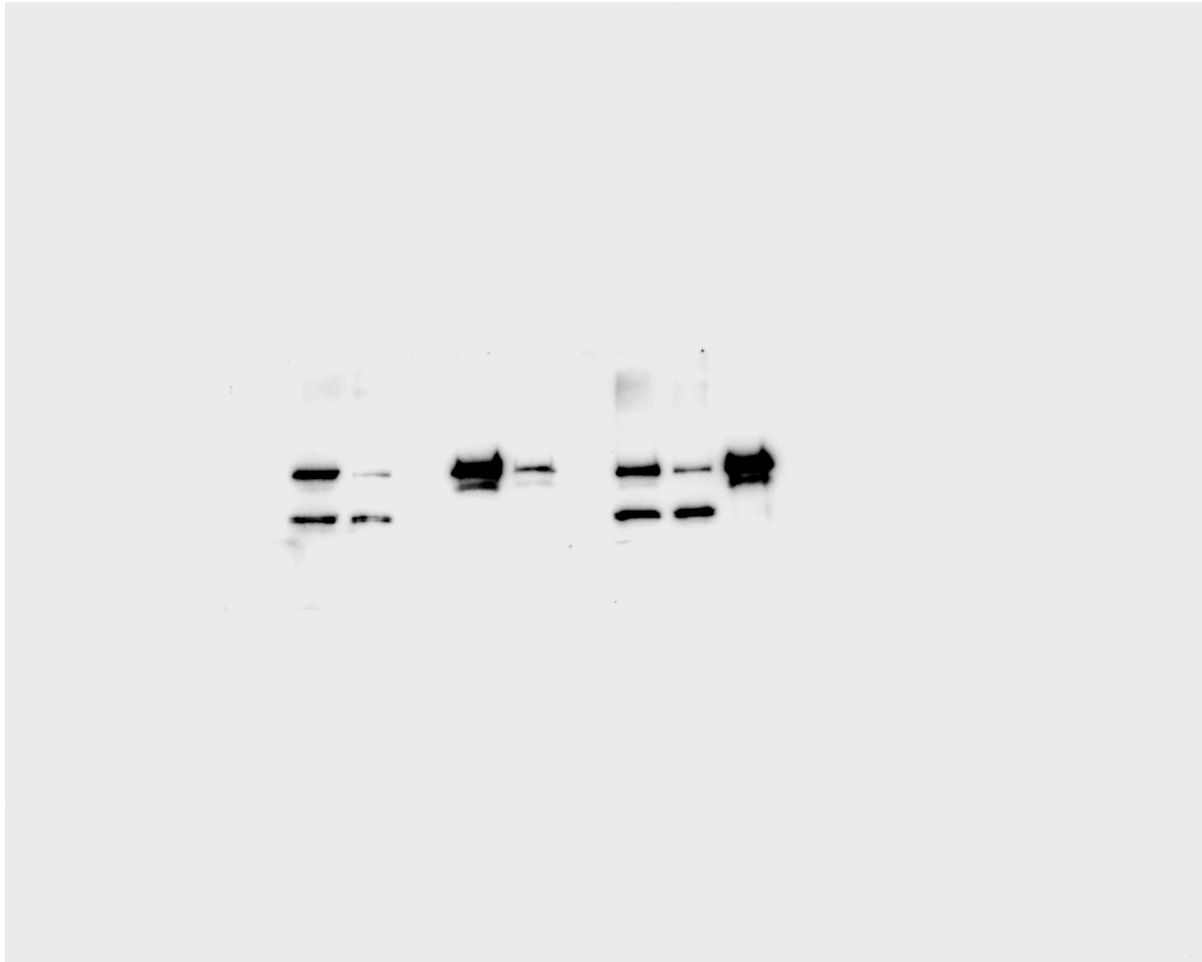

anti-SIP1

wild type

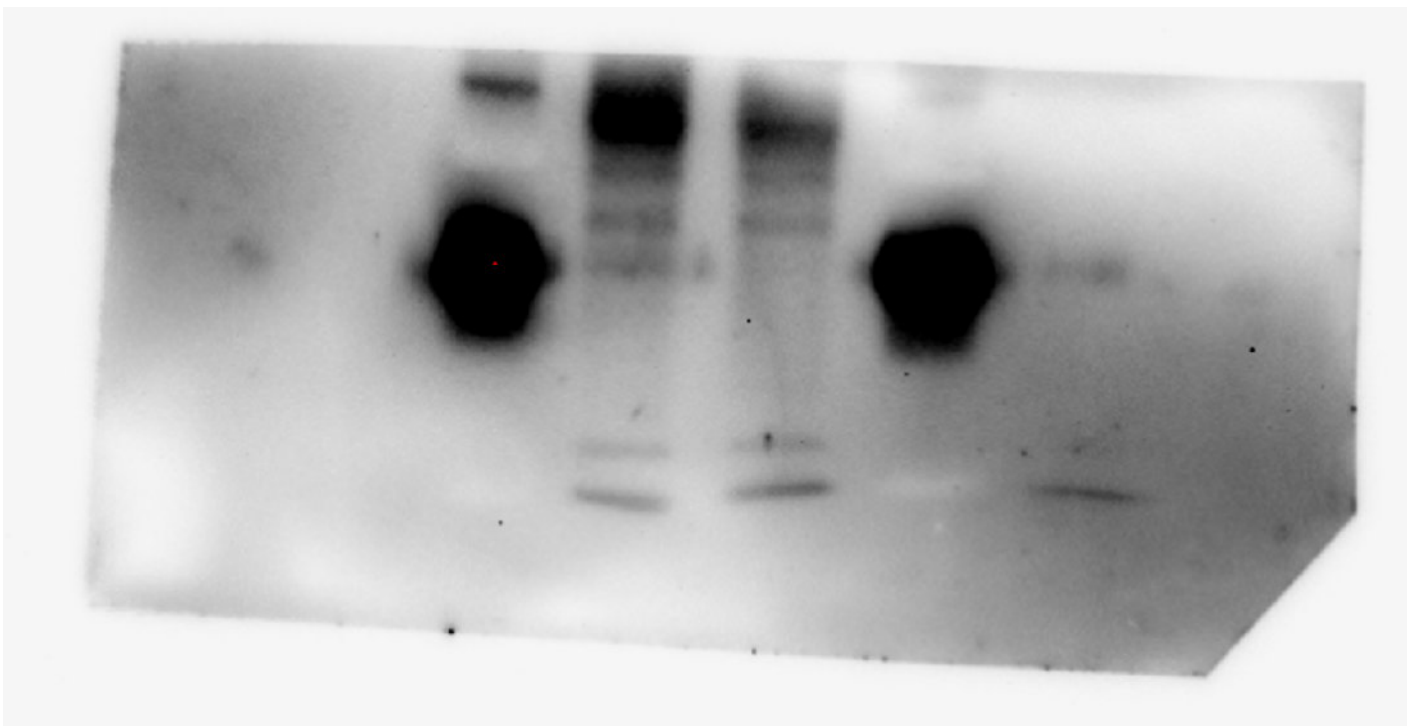

wrmScarlet::ALLO-1

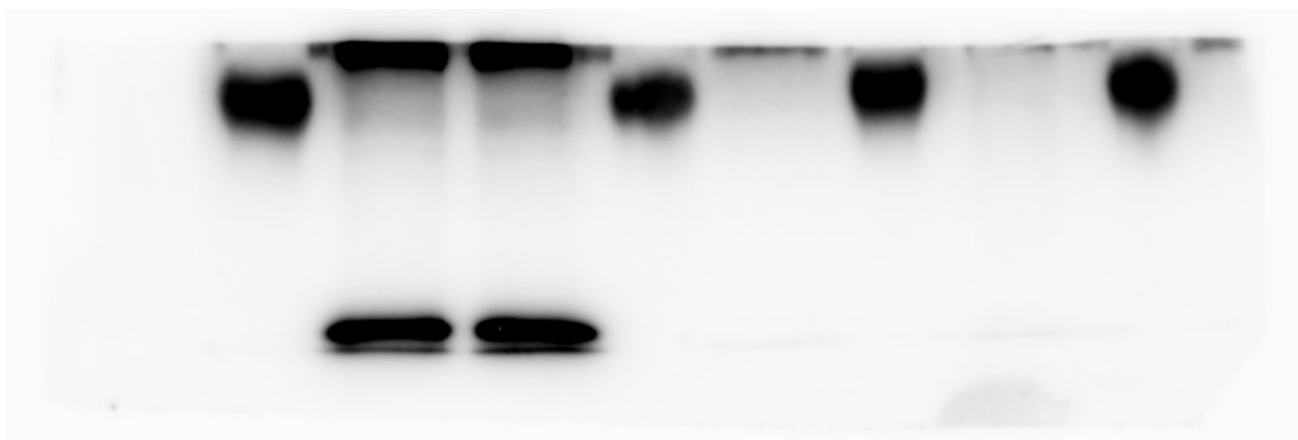

total protein

wild type

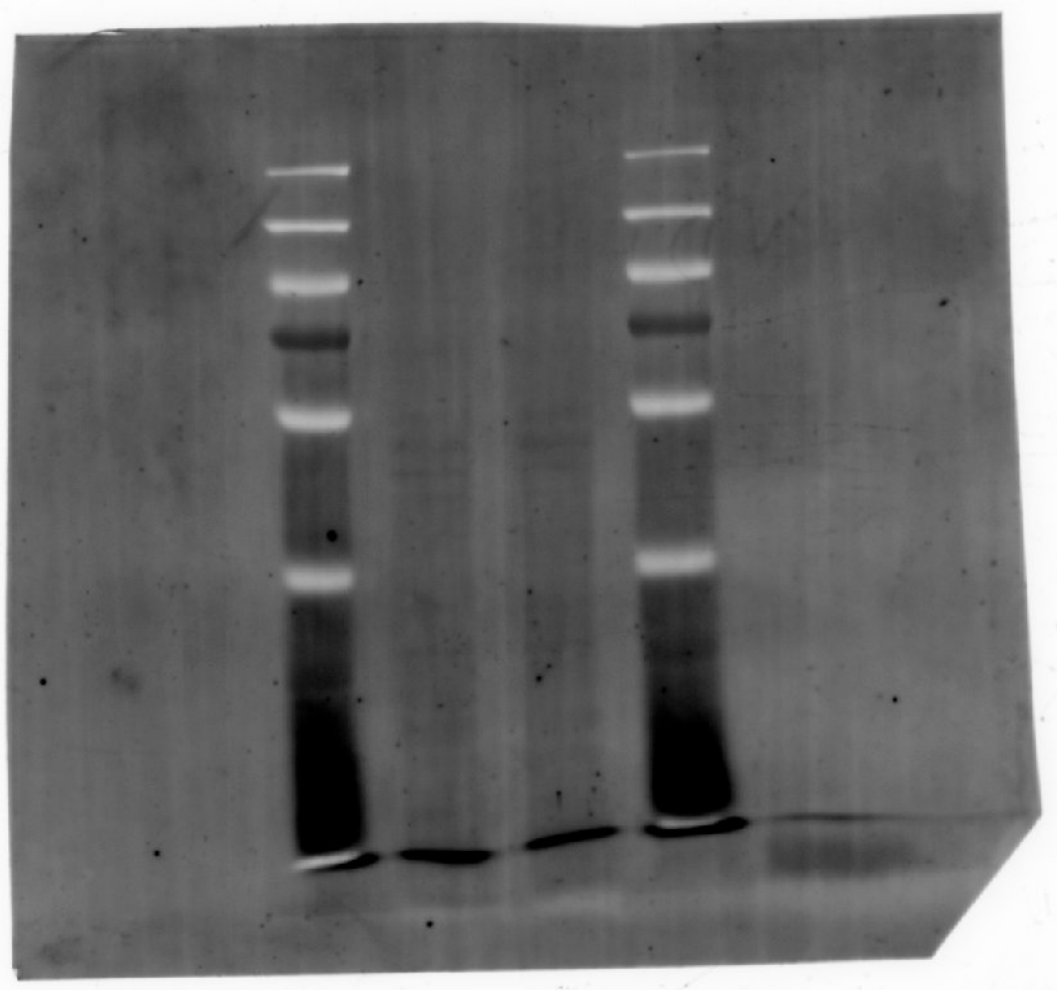

wrmScarlet::ALLO-1

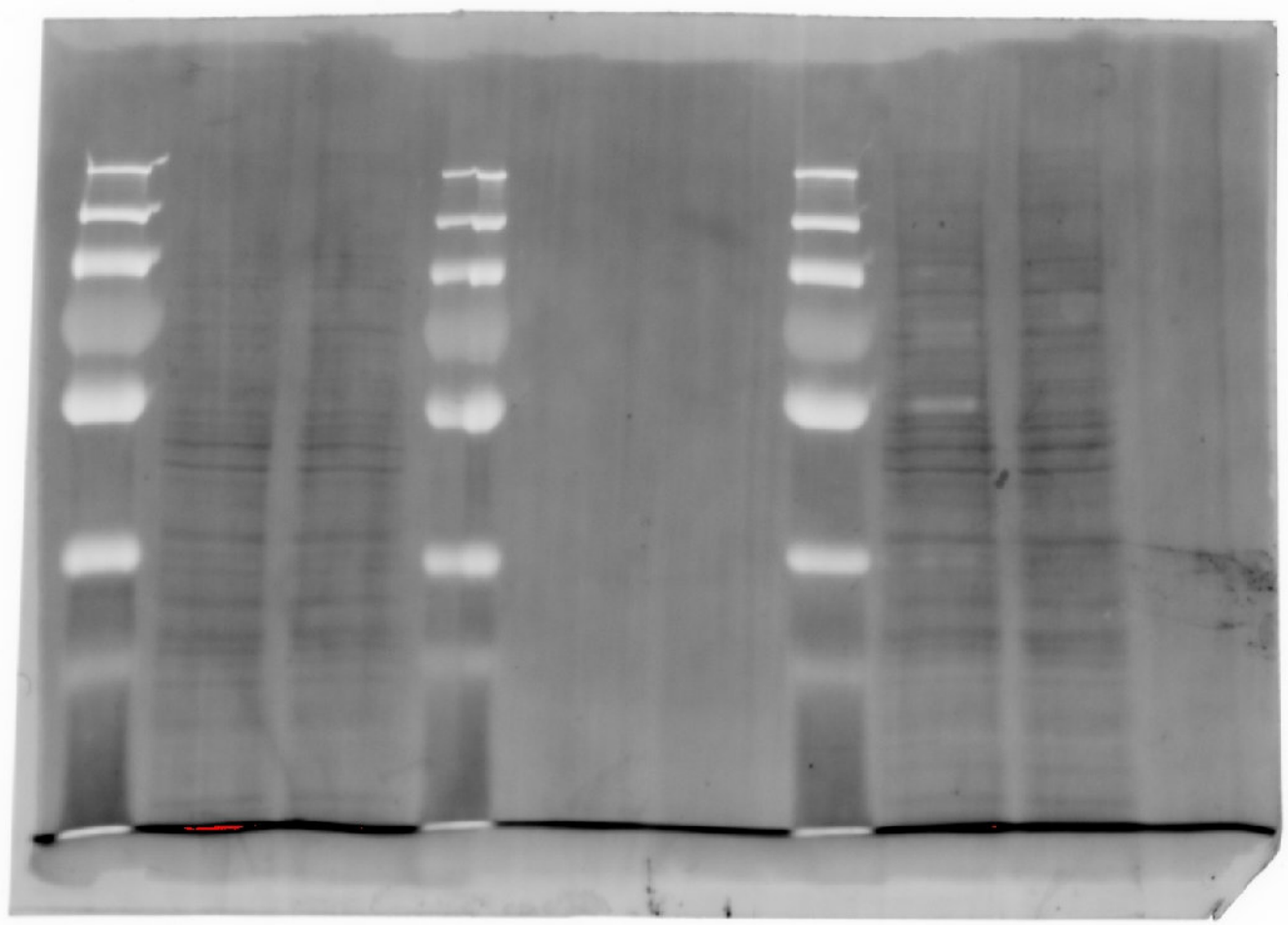

Uncropped immunoblot and total-protein loading control corresponding to Supplementary Fig. S4B

anti-FLAG

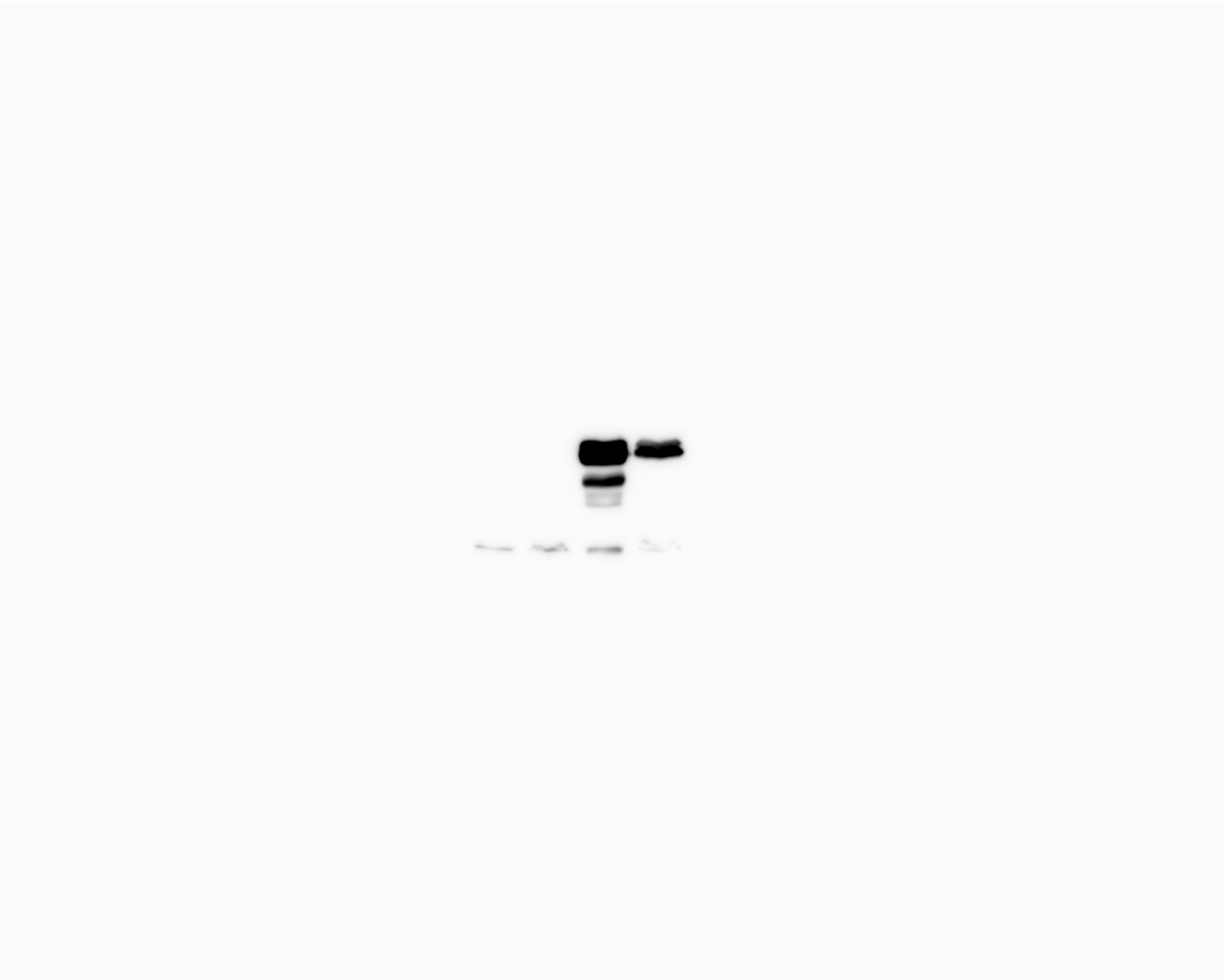

total protein

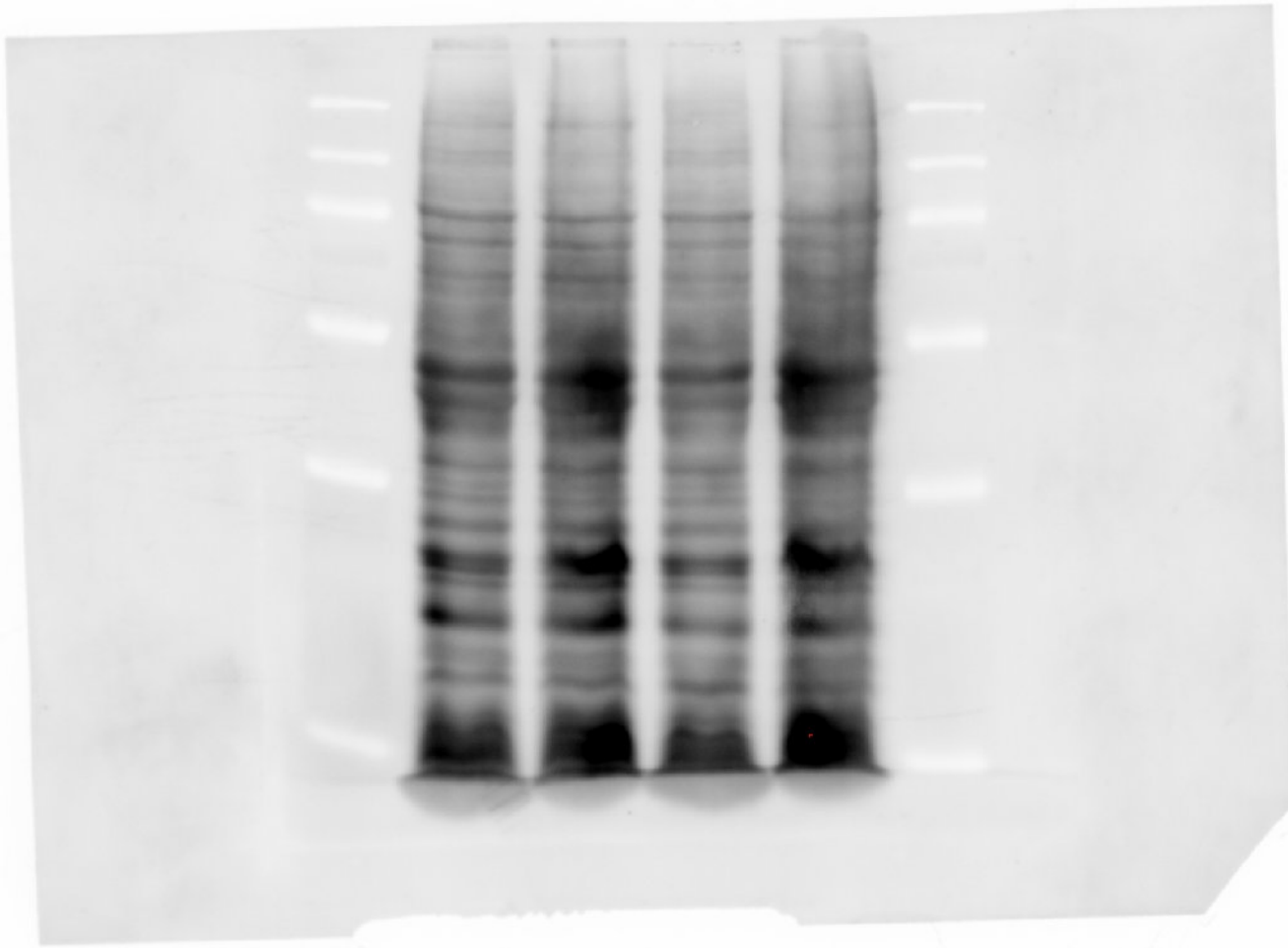

Supplement: Supplementary file 1 [file 42003_2026_11093_MOESM1_ESM.pdf]
