# Supplementary material for: An ALLO-1–associated network links mitochondrial quality control to functional recovery in structurally compromised muscle
Source: Commun Biol. 2026 Oct 6;9:1348. doi: 10.1038/s42003-026-11093-z (PMC13669348; doi:10.1038/s42003-026-11093-z)
Supplement: Supplementary file 2 [file 42003_2026_11093_MOESM2_ESM.pdf]

Description of Additional Supplementary Files

Supplementary Data 1. Tandem mass tag-based quantitative proteomics comparing wild type (N2) and *unc-45(m94)* animals across baseline, paralysis, and two recovery conditions. These correspond to matched recovery time points at which approximately 50% of the population had regained coordinated motility after return from the restrictive temperature. The table contains log2-transformed, median-normalized intensities for individual samples, together with the corresponding statistical results for each pairwise comparison (permutation-based two-sample *t*-test, S_0 = 0.1, FDR < 0.05). For each comparison, the table reports whether a protein passed the significance threshold, as well as the corresponding $-\log_{10}$ *P* value, q value, and $\log_2$ difference. The 12 comparisons include genotype comparisons at matched conditions, within-genotype comparisons between recovery and the corresponding reference condition, and cross-condition comparisons within unc-45(m94). Specifically, these are: (a) *unc-45(m94)* 25°C vs N2 25°C, (b) *unc-45(m94)* recovery 25°C vs N2 recovery 25°C, (c) N2 recovery 25°C vs N2 25°C, (d) *unc-45(m94)* recovery 25°C vs *unc-45(m94)* 25°C, (e) *unc-45(m94)* 15°C vs N2 15°C, (f) *unc-45(m94)* recovery 15°C vs N2 recovery 15°C, (g) N2 recovery 15°C vs N2 15°C, (h) *unc-45(m94)* recovery 15°C vs *unc-45(m94)* 15°C, (i) *unc-45(m94)* 25°C vs *unc-45(m94)* 15°C, (j) *unc-45(m94)* recovery 25°C vs *unc-45(m94)* recovery 15°C, (k) *unc-45(m94)* recovery 15°C vs *unc-45(m94)* 15°C, and (l) *unc-45(m94)* recovery 25°C vs *unc-45(m94)* 25°C.

Supplementary Data 2. Tandem mass tag-based quantitative proteomics identifying ALLO-1 associated proteins.

Supplementary Data 3. Tandem mass tag-based quantitative proteomics comparing the proteomes of wild type and muscle ALLO-1a worms.
