# Supplementary material for: An ALLO-1–associated network links mitochondrial quality control to functional recovery in structurally compromised muscle
Source: Commun Biol. 2026 Oct 6;9:1348. doi: 10.1038/s42003-026-11093-z (PMC13669348; doi:10.1038/s42003-026-11093-z)
Supplement: Supplementary file 3 [file 42003_2026_11093_MOESM6_ESM.pdf]

nature portfolio

Corresponding author(s): Wojciech Pokrzywa

Last updated by author(s): 14.09.2025

Reporting Summary

Nature Portfolio wishes to improve the reproducibility of the work that we publish. This form provides structure for consistency and transparency in reporting. For further information on Nature Portfolio policies, see our Editorial Policies and the Editorial Policy Checklist.

Statistics

For all statistical analyses, confirm that the following items are present in the figure legend, table legend, main text, or Methods section.

n/a	Confirmed	
☐	☒	The exact sample size (n) for each experimental group/condition, given as a discrete number and unit of measurement
☐	☒	A statement on whether measurements were taken from distinct samples or whether the same sample was measured repeatedly
☐	☒	The statistical test(s) used AND whether they are one- or two-sided<br>*Only common tests should be described solely by name; describe more complex techniques in the Methods section.*
☒	☐	A description of all covariates tested
☐	☒	A description of any assumptions or corrections, such as tests of normality and adjustment for multiple comparisons
☐	☒	A full description of the statistical parameters including central tendency (e.g. means) or other basic estimates (e.g. regression coefficient) AND variation (e.g. standard deviation) or associated estimates of uncertainty (e.g. confidence intervals)
☐	☒	For null hypothesis testing, the test statistic (e.g. F, t, r) with confidence intervals, effect sizes, degrees of freedom and P value noted<br>*Give P values as exact values whenever suitable.*
☒	☐	For Bayesian analysis, information on the choice of priors and Markov chain Monte Carlo settings
☐	☒	For hierarchical and complex designs, identification of the appropriate level for tests and full reporting of outcomes
☐	☒	Estimates of effect sizes (e.g. Cohen's d, Pearson's r), indicating how they were calculated

Our web collection on statistics for biologists contains articles on many of the points above.

Software and code

Policy information about availability of computer code

Data collection	Confocal images were acquired on a ZEISS LSM800; locomotion recordings were collected using the WormLab imaging system (MBF Bioscience). Additional acquisition systems were the Axio Zoom.V16/Axiocam 705 (Zeiss), Tecnai T12 BioTwin/TemCam-F416(R), ChemiDoc (Bio-Rad), Seahorse XFp (Agilent), and Q Exactive HF-X coupled to UltiMate 3000 nano-LC (Thermo Fisher Scientific).
Data analysis	GraphPad Prism v11.1.0; MaxQuant v1.6.17.0 with the Andromeda search engine; Perseus v1.6.10.0; ImageJ/Fiji with Mitochondria Analyzer v2.0.2 and JaCoP; ZEN (Zeiss); WormLab (MBF Bioscience); and WebGestalt for gene ontology enrichment. Proteomic searches used the UniProt C. elegans reference proteome UP000001940.

For manuscripts utilizing custom algorithms or software that are central to the research but not yet described in published literature, software must be made available to editors and reviewers. We strongly encourage code deposition in a community repository (e.g. GitHub). See the Nature Portfolio guidelines for submitting code & software for further information.

Data

Policy information about availability of data

All manuscripts must include a data availability statement. This statement should provide the following information, where applicable:

- Accession codes, unique identifiers, or web links for publicly available datasets
- A description of any restrictions on data availability
- For clinical datasets or third party data, please ensure that the statement adheres to our policy

The mass-spectrometry proteomics datasets generated during this study have been deposited in the ProteomeXchange Consortium via the PRIDE partner repository under accession numbers PXD069274, PXD069277 and PXD069441. The raw and source data supporting the findings, including data underlying quantitative analyses and figures, are available in Zenodo at https://doi.org/10.5281/zenodo.17371038.

Research involving human participants, their data, or biological material

Policy information about studies with human participants or human data. See also policy information about sex, gender (identity/presentation), and sexual orientation and race, ethnicity and racism.

Reporting on sex and gender	This study did not involve human participants, human data or human biological material.
Reporting on race, ethnicity, or other socially relevant groupings	*Please specify the socially constructed or socially relevant categorization variable(s) used in your manuscript and explain why they were used. Please note that such variables should not be used as proxies for other socially constructed/relevant variables (for example, race or ethnicity should not be used as a proxy for socioeconomic status). Provide clear definitions of the relevant terms used, how they were provided (by the participants/respondents, the researchers, or third parties), and the method(s) used to classify people into the different categories (e.g. self-report, census or administrative data, social media data, etc.) Please provide details about how you controlled for confounding variables in your analyses.*
Population characteristics	*Describe the covariate-relevant population characteristics of the human research participants (e.g. age, genotypic information, past and current diagnosis and treatment categories). If you filled out the behavioural & social sciences study design questions and have nothing to add here, write "See above."*
Recruitment	*Describe how participants were recruited. Outline any potential self-selection bias or other biases that may be present and how these are likely to impact results.*
Ethics oversight	*Identify the organization(s) that approved the study protocol.*

Note that full information on the approval of the study protocol must also be provided in the manuscript.

Field-specific reporting

Please select the one below that is the best fit for your research. If you are not sure, read the appropriate sections before making your selection.

☒ Life sciences ☐ Behavioural & social sciences ☐ Ecological, evolutionary & environmental sciences

For a reference copy of the document with all sections, see nature.com/documents/nr-reporting-summary-flat.pdf

Life sciences study design

All studies must disclose on these points even when the disclosure is negative.

Sample size	No statistical methods were used to predetermine sample size. Sample sizes were selected based on prior experience with the respective C. elegans assays, taking into account assay variability and practical feasibility. Their adequacy was assessed empirically through the consistency of results across independently prepared biological replicates. Unless otherwise indicated, experiments were independently repeated at least three times using biologically independent samples. Biological replicates were defined as independently prepared C. elegans populations processed separately from culture through sample preparation and analysis. Sample sizes and units of measurement varied by assay. Motility assays included three independent biological replicates, with at least 75 animals analysed per replicate. Sarcomere organisation was assessed in 35-50 confocal images per condition, with each image classified using predefined criteria. Mitochondrial morphology was quantified from individual muscle cells, analysing at least one region of interest (ROI) in a minimum of 20 images per condition across three biological replicates. Colocalisation analysis included 27-36 ROIs representing individual cells per condition. Transmission electron microscopy analyses included 14 animals per condition; measurements from 2-5 muscle cells were averaged for each animal, and each animal contributed a single value to the statistical analysis. For the mitoRosella analyses shown in Figure 5A,C, 20 images were analysed per condition per biological replicate across three independent biological replicates. PolyQ40::YFP aggregation was quantified in 30 animals across three independent biological replicates. Brood-size assays included 10 animals per biological replicate across three independent biological replicates, with two animals housed per plate and reproductive output recorded per plate. Seahorse assays used approximately 10–15 animals per well, with oxygen-consumption rates normalised to the number of animals in each well.
Data exclusions	In the ALLO-1 co-immunoprecipitation proteomics analysis, quality assessment by principal component analysis identified one control replicate (ctrl_1) with an atypical profile consistent with a technical issue. This sample was omitted from downstream statistical comparisons;

all three wrmScarlet::ALLO-1 immunoprecipitation replicates were retained and compared with the two remaining control replicates. All original data, including the omitted control replicate, are provided in Supplementary Dataset 2 and deposited in the public repository. Standard proteomic preprocessing removed reverse hits, potential contaminants, entries identified only by site, and protein groups lacking complete quantitative data across TMT channels.

Replication

Unless otherwise indicated, experiments were independently repeated at least three times using biologically independent samples. Representative microscopy images and immunoblots are reported to represent at least three independent biological experiments. The manuscript defines biological replicates as independently prepared worm populations processed separately through culture, sample preparation and analysis.

Randomization

No formal randomization was performed. Age-synchronised C. elegans were assigned to experimental groups according to genotype and treatment. Animals were analysed at matched developmental stages, and culture conditions, handling and assay procedures were kept consistent between comparison groups, apart from the experimental variables under investigation.

Blinding

Confocal images used for qualitative assessment of sarcomere organisation were scored blind using predefined organised/disorganised categories. Quantitative analysis of mitochondrial morphology using the TOMM-20::GFP reporter was also performed blind to experimental group.

Reporting for specific materials, systems and methods

We require information from authors about some types of materials, experimental systems and methods used in many studies. Here, indicate whether each material, system or method listed is relevant to your study. If you are not sure if a list item applies to your research, read the appropriate section before selecting a response.

Materials & experimental systems

n/a	Involved in the study	
☐	☒	Antibodies
☒	☐	Eukaryotic cell lines
☒	☐	Palaeontology and archaeology
☐	☒	Animals and other organisms
☒	☐	Clinical data
☒	☐	Dual use research of concern
☒	☐	Plants

Methods

n/a	Involved in the study	
☒	☐	ChIP-seq
☒	☐	Flow cytometry
☒	☐	MRI-based neuroimaging

Antibodies

Antibodies used

Anti-SIP-1: provided by Prof. Johannes Buchner, Technical University of Munich; cited as Fleckenstein et al., Molecular Cell 58, 1067-1078 (2015), manuscript reference 18. Used for immunostaining at 1:250; also used to detect SIP-1 in ALLO-1 immunoprecipitates as described in the Results. Anti-mCherry: Abcam, cat. ab167453, used for immunoblotting. Anti-FLAG: Sigma-Aldrich, cat. F3165, used for immunoblotting. HRP-conjugated secondary antibodies: Sigma-Aldrich, cats. A0545 and A9044, both at 1:10,000. Immunostaining secondary antibody: Thermo Fisher Scientific, reported as cat. A212452, at 1:500. RFP-Trap Magnetic Agarose: ChromoTek, cat. rtma-20; 25 microlitres of slurry per sample for wrmScarlet::ALLO-1 immunoprecipitation. Untagged animals were processed in parallel as controls.

Validation

The manuscript identifies the anti-SIP-1 reagent by its published source (reference 18). Untagged control animals were used in parallel ALLO-1 immunoprecipitations; a faint SIP-1 signal was reported in tagged immunoprecipitates but not in the untagged controls. This supports interpretation of the co-immunoprecipitation, but does not by itself establish antibody specificity for every application.

Animals and other research organisms

Policy information about studies involving animals; ARRIVE guidelines recommended for reporting animal research, and Sex and Gender in Research

Laboratory animals

Laboratory C. elegans were maintained on NGM seeded with E. coli OP50 or HT115 at 15, 20 or 25 degrees C, depending on the assay. Key strains included Bristol N2 and unc-45(m94) (CGC); allo-1(syb8353[wrmScarlet::allo-1]) and allo-1(syb10325) (SunyBiotech); integrated muscle FLAG-ALLO-1a (SunyBiotech, PHX8969); the wacIs14 muscle TOMM-20::GFP reporter; mitoRosella; pdr-1(tm395); and the crosses listed in Methods. Age-synchronised animals were generally treated from L4 and analysed as young adults. Recovery experiments used 15 degrees C, 18-20 h at 25 degrees C, and return to 15 degrees C.

Wild animals

The study used laboratory-maintained C. elegans strains; no wild animals were used in the experiments described.

Reporting on sex

The recovery, brood-size, cyclosporin A and paraquat Methods explicitly specify age-synchronised L4 hermaphrodites. No male-specific experiments or comparisons between sexes are described.

Field-collected samples | No field-collected samples were used in the study described.

Ethics oversight | No ethical approval was required for the experiments involving the nematode Caenorhabditis elegans.

Note that full information on the approval of the study protocol must also be provided in the manuscript.

Plants

Seed stocks | *Report on the source of all seed stocks or other plant material used. If applicable, state the seed stock centre and catalogue number. If plant specimens were collected from the field, describe the collection location, date and sampling procedures.*

Novel plant genotypes | *Describe the methods by which all novel plant genotypes were produced. This includes those generated by transgenic approaches, gene editing, chemical/radiation-based mutagenesis and hybridization. For transgenic lines, describe the transformation method, the number of independent lines analyzed and the generation upon which experiments were performed. For gene-edited lines, describe the editor used, the endogenous sequence targeted for editing, the targeting guide RNA sequence (if applicable) and how the editor was applied.*

Authentication | *Describe any authentication procedures for each seed stock used or novel genotype generated. Describe any experiments used to assess the effect of a mutation and, where applicable, how potential secondary effects (e.g. second site T-DNA insertions, mosiacism, off-target gene editing) were examined.*
